# The Evolution of Duplicated Genes of the Cpi-17/Phi-1 (*ppp1r14*) Family of Protein Phosphatase 1 Inhibitors in Teleosts

**DOI:** 10.3390/ijms21165709

**Published:** 2020-08-09

**Authors:** Irene Lang, Guneet Virk, Dale C. Zheng, Jason Young, Michael J. Nguyen, Rojin Amiri, Michelle Fong, Alisa Arata, Katia S. Chadaideh, Susan Walsh, Douglas C. Weiser

**Affiliations:** 1 Department of Biological Sciences, University of the Pacific, Stockton, CA 98211, USA; i_lang@u.pacific.edu (I.L.); g_virk@u.pacific.edu (G.V.); dzheng@pacific.edu (D.C.Z.); j_young5@u.pacific.edu (J.Y.); m_nguyen32@u.pacific.edu (M.J.N.); r_amiri@u.pacific.edu (R.A.); m_fong8@u.pacific.edu (M.F.); a_arata@u.pacific.edu (A.A.); kchadaideh@g.harvard.edu (K.S.C.); 2 Department of Human Evolutionary Biology, Harvard University, Cambridge, MA 02138, USA; 3 Life Sciences, Soka University of America, Aliso Viejo, CA 92656, USA; swalsh@soka.edu

**Keywords:** Danio rerio, Phi-1 (ppp1r14b), Cpi-17 (ppp1r14a), PP1, Mypt1, genome duplication

## Abstract

The Cpi-17 (*ppp1r14*) gene family is an evolutionarily conserved, vertebrate specific group of protein phosphatase 1 (PP1) inhibitors. When phosphorylated, Cpi-17 is a potent inhibitor of myosin phosphatase (MP), a holoenzyme complex of the regulatory subunit Mypt1 and the catalytic subunit PP1. Myosin phosphatase dephosphorylates the regulatory myosin light chain (Mlc2) and promotes actomyosin relaxation, which in turn, regulates numerous cellular processes including smooth muscle contraction, cytokinesis, cell motility, and tumor cell invasion. We analyzed zebrafish homologs of the Cpi-17 family, to better understand the mechanisms of myosin phosphatase regulation. We found single homologs of both Kepi (*ppp1r14c*) and Gbpi (*ppp1r14d*) in silico, but we detected no expression of these genes during early embryonic development. Cpi-17 (*ppp1r14a*) and Phi-1 (*ppp1r14b*) each had two duplicate paralogs, (*ppp1r14aa* and *ppp1r14ab*) and (*ppp1r14ba* and *ppp1r14bb*), which were each expressed during early development. The spatial expression pattern of these genes has diverged, with *ppp1r14aa* and *ppp1r14bb* expressed primarily in smooth muscle and skeletal muscle, respectively, while *ppp1r14ab* and *ppp1r14ba* are primarily expressed in neural tissue. We observed that, in in vitro and heterologous cellular systems, the Cpi-17 paralogs both acted as potent myosin phosphatase inhibitors, and were indistinguishable from one another. In contrast, the two Phi-1 paralogs displayed weak myosin phosphatase inhibitory activity in vitro, and did not alter myosin phosphorylation in cells. Through deletion and chimeric analysis, we identified that the difference in specificity for myosin phosphatase between Cpi-17 and Phi-1 was encoded by the highly conserved PHIN (phosphatase holoenzyme inhibitory) domain, and not the more divergent N- and C- termini. We also showed that either Cpi-17 paralog can rescue the knockdown phenotype, but neither Phi-1 paralog could do so. Thus, we provide new evidence about the biochemical and developmental distinctions of the zebrafish Cpi-17 protein family.

## 1. Introduction

Teleosts are a large infraclass of ray-finned fish (class Actinopterygii), with 30,000 species, that comprise approximately 95% of fish and 50% of vertebrate species [1]. Certain teleosts, such as medaka (*Oryzias latipes*) and zebrafish (*Danio rerio*), have become important genetic model organisms of vertebrate development and human disease, which allows researchers to link fish and human gene functions. These genetic models have been complicated by several rounds of whole genome duplication (WGD) during vertebrate evolution, two of which occurred before the divergence of teleosts and tetrapods (vertebrate genome duplication; VGD1 and VGD2), and a teleost specific duplication (TGD), which occurred after the divergence of Semionotiforms (gars) and Amiiformes (bowfin) [2,3,4,5,6]. Additional duplications have occurred in teleosts, including a salmonid-specific (SaGD) whole genome duplication [7,8]. As a result, the large number of ohnologs (paralog pairs produced by genome duplication) [9], the lineage specific loss of certain ohnologs, and the asymmetric evolution of the pairs of ohnologs have complicated ortholog identification. The most common fate of duplicated genes is loss-of-function (pseudogenization) from the accumulation of deleterious mutations, which can occur in all teleosts, or in a lineage specific manner [10]. In addition, duplicated genes can remain redundant, acquire new functions not seen in the ancestral gene, or subfunctionalize by splitting the ancestral gene’s function [11]. This subfunctionalization can occur due to changes in the activity of the protein product, or by changes in gene expression of the paralogs [11].

In this work, we have focused on the evolution and function of the vertebrate specific Cpi-17 (*ppp1r14*) family of protein phosphatase 1 (PP1) inhibitors [12]. PP1 is an abundant and ubiquitously expressed serine-threonine phosphatase that regulates diverse cellular processes including actomyosin contractility, glycogen metabolism, cell cycle, gene expression, protein synthesis, and neuronal signaling [13]. The purified PP1 catalytic subunit is highly promiscuous, and will dephosphorylate a vast array of phosphoproteins [13]. In the cell, however, a suite of regulatory and inhibitory subunits precisely controls PP1 activity [14,15]. Regulatory subunits bind to PP1 and can target PP1 to a subset of substrates, inhibit PP1 toward alternate substrates, control PP1 subcellular localization, and bring the assembled holoenzyme form of PP1 under the control of various upstream signaling pathways [13,14,15]. The Cpi-17 family is unique among PP1 inhibitors in its high potency toward holoenzyme complexes of PP1 [12]. Most PP1 inhibitors directly compete with regulatory subunits for PP1 binding, and are much more potent against the isolated catalytic subunit [16]. Some PP1 inhibitors form tripartite complexes with PP1 and regulators, allowing them to inhibit holoenzymes [17,18,19]. The Cpi-17 family does not compete with regulatory subunits; instead, phosphorylated Cpi-17 binds PP1 in the active site to block substrate binding [20,21]. 

One of the primary cellular targets of the Cpi-17 family is myosin phosphatase (MP), a holoenzyme complex of the regulatory subunit Mypt1 and the catalytic subunit PP1 [22,23]. MP dephosphorylates myosin light chain (Mlc2), resulting in actomyosin relaxation [24] and opposes the action of numerous Mlc2 kinases, including myosin light chain kinase (Mlck), zipper-interacting protein kinase (Zipk), and Rho-associated protein kinases (Rock) [25]. Precise control of Mlc2 phosphorylation is required for numerous cellular processes, including smooth muscle contraction, cytokinesis, cell motility and tumor cell invasion [26]. Cpi-17 family members themselves are phosphorylated by kinases including PKC (protein kinase C), ILK (integrin-linked kinase), and Rock (Rho-associated protein kinase), and play a key role in placing actomyosin contractility under the control of G-protein coupled receptors and other upstream signals [27,28,29,30,31]. 

Zebrafish has emerged as an excellent model to study the role of MP during early vertebrate development. Mutation or knockdown of zygotic Mypt1 (*ppp1r12a*) is embryonic lethal, resulting in the failure of liver development, disorganized somites, excessive cellular contractility of the neural epithelium, and motor axonogenesis [32,33,34,35]. If both maternal and zygotic *ppp1r12a* are knocked down, embryos develop hypercontractile mesodermal cells that fail to undergo proper morphogenetic cell movement, including the convergent extension (CE) of the presomitic mesoderm during gastrulation [36,37]. CE is a cellular process by which cells first migrate towards the future dorsal side of the embryo, and subsequently intercalate between neighboring cells, resulting in an overall dorsal-ventral narrowing (convergence) and anterior–posterior lengthening (extension) of the embryonic tissue [38,39,40]. CE is highly sensitive to changes in Mlc2 phosphorylation, and can be disrupted by overexpression or knockdown of Mlc2 kinases or phosphatases [36,41]. We hypothesized that the Cpi-17 family would regulate MP function during early development in zebrafish. 

Mammals have four genes in the Cpi-17 family; Cpi-17 (PPP1R14A), Phi-1 (PPP1R14B), KEPI (PPP1R14C), and GBPI (PPP1R14D) [12]. We found single orthologs of both KEPI (*ppp1r14c*) [42] and GBP1 (*ppp1r14d*) [43] in silico, but we detected no expression of these genes during early embryonic development. We found two duplicates of Cpi-17 (*ppp1r14aa* and *ppp1r14ab*) and Phi-1 (*ppp1r14ba* and *ppp1r14bb*), which appeared to be the products of the teleost WGD. Phylogenetic analysis indicated that many species of teleost have two copies of *ppp1r14a*, while some have lost one paralog. Some teleosts have also retained duplicate *ppp1r14b* paralogs, but most teleost lineages have lost *ppp1r14ba,* retaining a single copy. In mammals, Cpi-17 is widely expressed, but is enriched in tonic muscles, such as arteries, and the degree of PKC-mediated smooth muscle contraction correlates strongly with Cpi-17 abundance [44]. Cpi-17 is also enriched in the central nervous system, and plays a critical role in cerebellar long-term synaptic depression [45]. In mammals, Phi-1 is broadly expressed [46]. In zebrafish, the paralog pairs have diverged in expression patterns, with *ppp1r14aa* and *pppr14bb* expressed most strongly in muscle tissue [47,48,49], while *ppp1r14ab* and *ppp1r14ba* are expressed most strongly in neural tissue [49,50]. This evolutionary pattern is common after WGD. We then set out to determine if the paralog pairs diverged in biochemical function. We found that both Cpi-17 paralogs were equally potent inhibitors of the MP, in in vitro and heterologous cell systems. In contrast, both Phi-1 paralogs were weaker inhibitors in vitro and did not alter Mlc2 phosphorylation in cellular systems. Using structure-function analysis, we determined that the greater potency of Cpi-17 was dependent on the conserved PHIN (phosphatase holoenzyme inhibitory) domain [51]. The only published loss-of-function phenotype for a Cpi-17 family member in zebrafish is that *ppp1r14ab* is required for proper gene expression in rhombomere 4 of the hindbrain [50]. We observed that either Cpi-17 paralog but not the Phi-1 paralogs could rescue the *ppp1r14ab* morphant embryos. In conclusion, we observed that Cpi-17 and Phi-1 each have duplicated paralogs, and though the paralogs have diverged in expression, their cellular function has remained the same.

## 2. Results and Discussion

### 2.1. Identification of the Zebrafish ppp1r14 Gene Family

**Figure 1:**
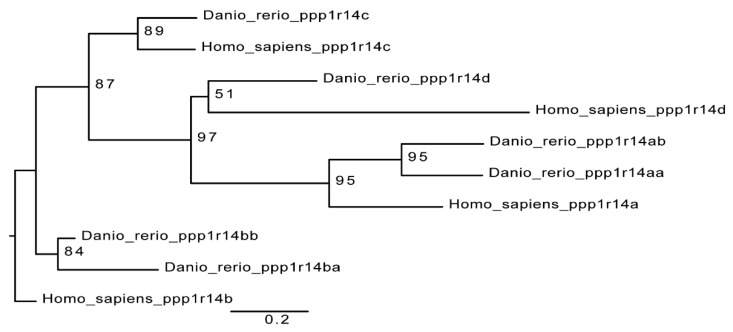
Cpi-17 gene family in zebrafish. The Cpi-17 family in humans consists of *ppp1r14a*, *ppp1r14b*, *ppp1r14c*, and *ppp1r14d*. The Cpi-17 family in zebrafish consists of two paralogs of both *ppp1r14a* and *ppp1r14b*, and a single copy of *ppp1r14c* and *ppp1r14d*. *Ppp1r14c* and *ppp1r14d* were not expressed in zebrafish early embryonic development.

The zebrafish genome contains 6 members of the *ppp1r14* (Cpi-17) gene family; a pair of Cpi-17 genes, *ppp1r14aa* and *ppp1r14ab*, a pair of Phi-1 genes, *ppp1r14ba* and *ppp1r14bb*, a single Kepi ortholog, *ppp1r14c,* and a single Gbpi ortholog, *ppp1r14d*. The mammalian gene family contains four genes, one each for Cpi-17, Phi-1, Kepi, and Gbpi [12]. A simple phylogenetic comparison of the PHIN domain of the zebrafish genes and the human genes is consistent with a model of whole genome duplication, leading to duplicated paralogs, some of which are retained in the genome, while others are lost (Figure 1). Primers were designed against all six zebrafish Cpi-17 gene members and semi-quantitative reverse transcriptase PCR was performed on mRNA isolated from early zebrafish embryos (from 2 hpf up to 5 dpf). Both paralogs of Cpi-17 and both paralogs of Phi-1 were expressed at various developmental stages, while no expression was detected for *ppp1r14c* or *ppp1r14d* during early development (data not shown). Thus, in order to better understand the regulation of myosin phosphatases during early zebrafish development, we focused our analysis on *ppp1r14aa*, *ab, ba,* and *bb*. 

**Figure 2:**
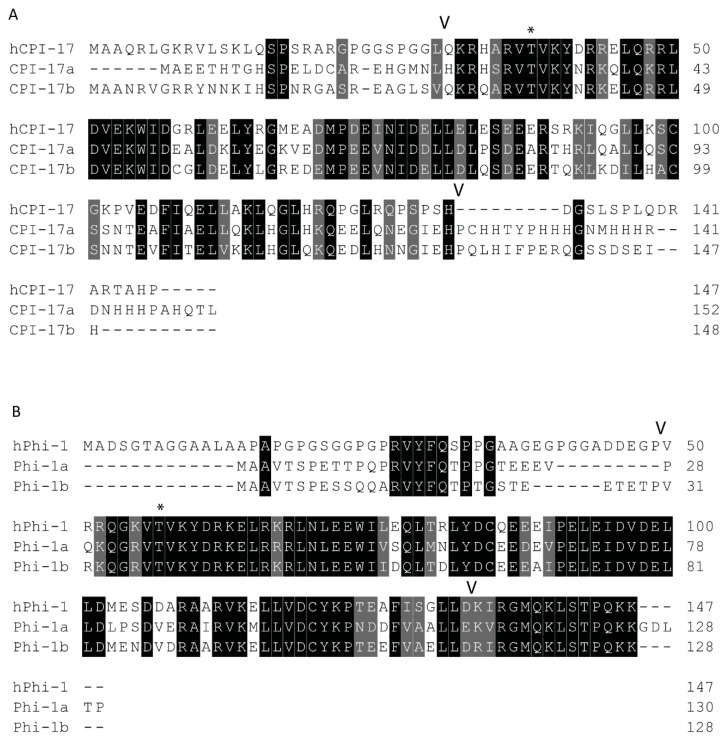
Protein sequences of zebrafish Cpi-17 and Phi-1 paralogs aligned to their human orthologs. (**A**). Protein alignment of zebrafish Cpi-17a and Cpi-17b with human Cpi-17. (**B**). Protein alignments of zebrafish Phi-1a and Phi-1b with human Phi-1. The conserved 86-residue long conserved PHIN domain is marked with flanking V, with inhibitory phosphorylation sites T31 (Cpi-17a), T37 (Cpi-17b), T35 (Phi-1a), and T38 (Phi-1b) indicated by *.

Both paralogs of *ppp1r14a* have similar simple 4-exon genomic organization, with a single splice variant with 3 short exons and 1 long exon. Both *ppp1r14b* paralogs also have four exons. Four transcript variants are observed for *ppp1r14ba* that all produce the same protein product, differing only in the transcriptional start site and the length of a noncoding exon. Two transcript variants of *ppp1r14bb* also produce the same protein product. The protein sequences of Cpi-17 and Phi-1 show considerable conservation of the central PHIN domain (CPI-17a and Phi-1b are 30% identical and 46% similar within the PHIN domain), while the N and C-termini of Cpi-17 are more divergent (Figure 2). Human Cpi-17 has a critical threonine at residue 38, which when phosphorylated, can inhibit PP1. This phosphorylation site is conserved in both Cpi-17a (T31) and Cpi-17b (T37) (Figure 2A). In human Cpi-17, Y41 and R43 are essential for PP1 inhibition [52], and are conserved in both Cpi-17a and Cpi-17b. R44 and D42 are also required for PP1 inhibition [52], but are substituted for a K and N in both zebrafish paralogs. Relatively little sequence homology is observed in the N or C termini of the Cpi-17 paralogs. Zebrafish Phi-1a and Phi-1b both show considerable sequence conservation, with each other and with the human Phi-1 (Figure 2B). The critical phosphorylation site T57 is conserved in both zebrafish Phi-1a and Phi-1b, along with nearly all flanking residues, including the critical Y60. Unlike Cpi-17, large blocks of homology are found in both the N and C termini of Phi-1 outside of the PHIN domain. In the N-termini, a putative PP1-binding domain with the sequence RVYF is found, while the C-termini has a large block of conserved amino acids of uncharacterized function. Taken together, these data indicate a high degree of homology between human and teleost Cpi-17 and Phi-1 proteins, and we hypothesized that they retain similar biochemical function.

### 2.2. Synteny and Phylogenetic Analysis of Duplicated ppp1r14 Genes

**Figure 3:**
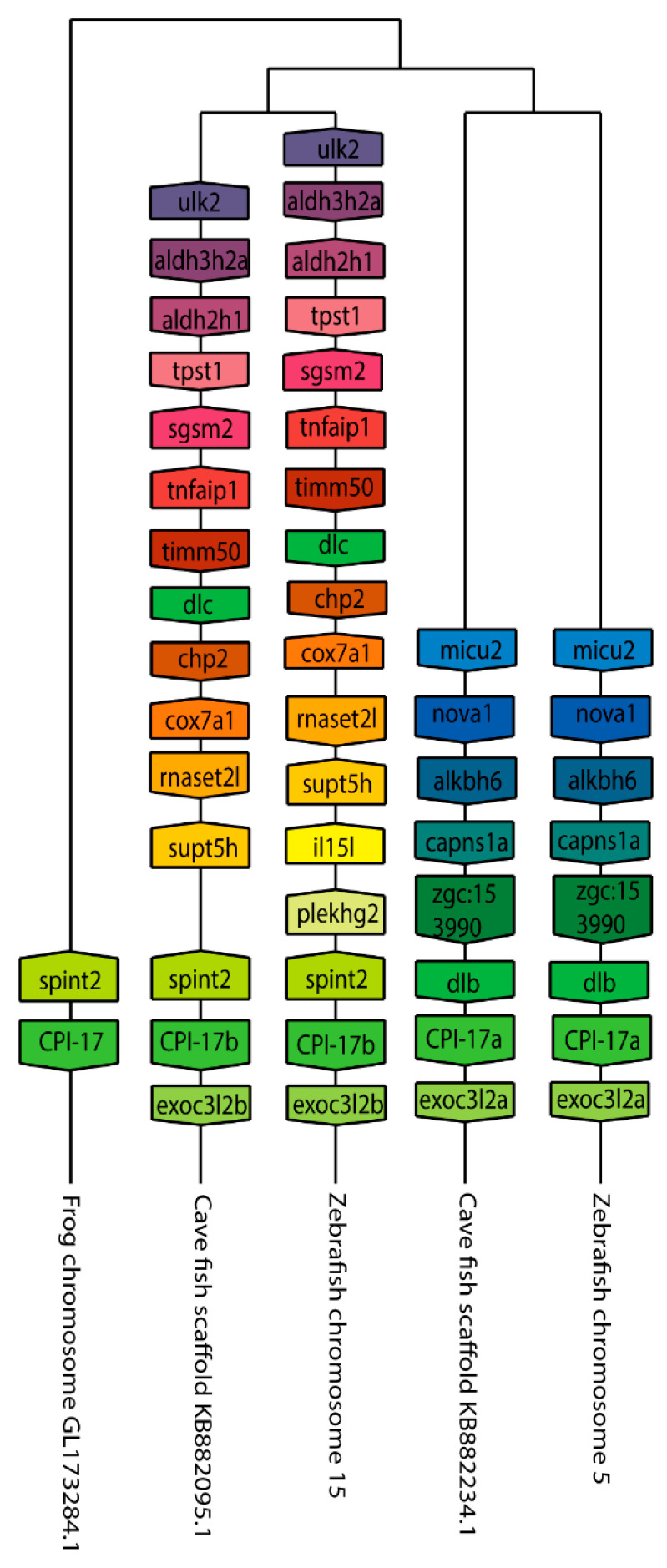
The genomic organization of the Cpi-17 (*ppp1r14a)* paralogs in zebrafish. Genomicus was used to analyze Cpi-17 (*ppp1r14a)* synteny between *Danio rerio (Zebrafish)*, and the most closely related sequenced relative, *Astyanax mexicanus (Cavefish)*. *Xenopus laevis (African clawed frog)* was used as an outgroup. Homologous genes are shown with matching coloration, and the gene direction on the chromosome is indicated by the direction of the boxed arrow. Species and chromosome numbers are noted.

**Figure 4:**
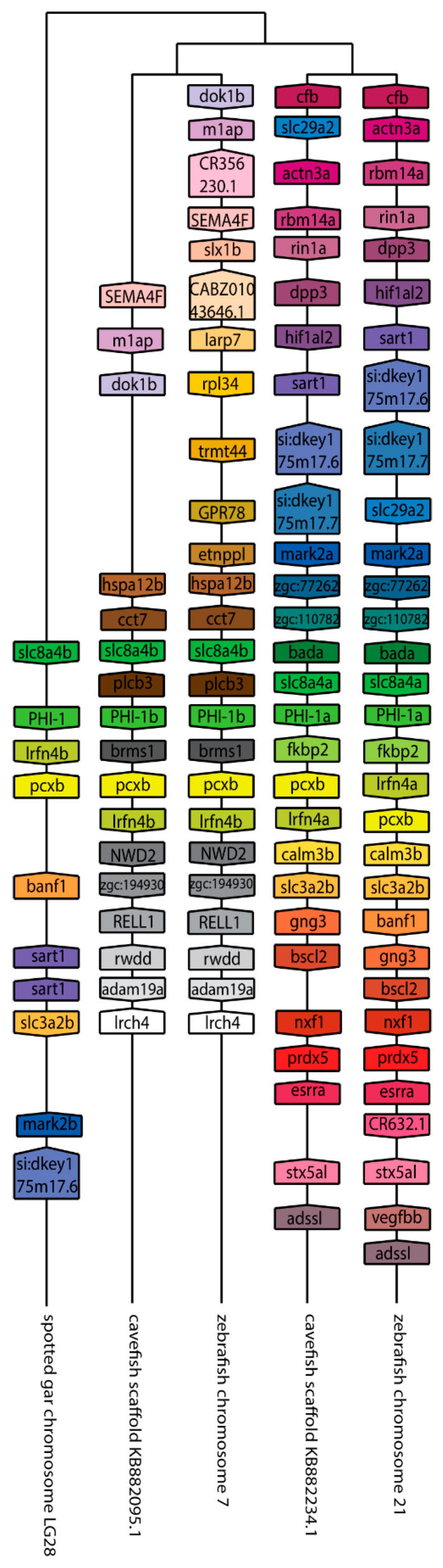
The genomic organization of the *ppp1r14b* paralogs in zebrafish. Genomicus was used to analyze *ppp1r14b* synteny between *Danio rerio* (zebrafish), and the most closely related sequenced relative, *Astyanax mexicanus (cavefish)*. *Lepisosteus oculatus* (spotted gar) was used as an outgroup. Homologous genes are shown with matching coloration, and the gene direction on the chromosome is indicated by the direction of the boxed arrow. Species and chromosome numbers are noted.

We next set out to better understand the evolution of the *ppp1r14* family by analyzing the genomic organization and synteny of the duplicated Cpi-17 and Phi-1 genes in teleosts, using the genomicus browser [53]. The zebrafish genes were compared to the cavefish *Astyanax mexicanus,* which like zebrafish, is a cypriniform and underwent the teleost WGD. *Xenopus laevis* was used as an outgroup for the *ppp1r14a* genes, and *Lepisosteus oculatus* was used for *ppp1r14b* genes; neither species’ genome underwent the teleost WGD. *Xenopus laevis* encodes *ppp1r14a* on chromosome 8, adjacent to the serine protease inhibitor *spint2* (Figure 3). Zebrafish *ppp1r14aa* is found on chromosome 5, while *ppp1r14ab* was encoded on chromosome 15. In both zebrafish and cavefish, the synteny with *spint2* was conserved with *ppp1r14aa*, but not with *ppp1r14ab*. The duplicated genes for *ppp1r14a* in both zebrafish and cavefish are flanked by multiple duplicated genes, including the delta genes (*dlb* and *dlc*) and the excocyst component-like genes *exco3l2a* and *exco3l2b*, each of which appear to be the product of the teleost WGD. Other flanking genes show considerable conservation in order and orientation between zebrafish and cavefish (Figure 3). In the gar *Lepisosteus oculatus, ppp1r14b* was found on the chromosome LG28 flanked by multiple genes, including the solute carrier family 8 member *slc8a4b*, the leucine rich repeat and fibronectin type III domain containing *irfn4b,* and the pyruvate carboxylase *pcxb* (Figure 4). The duplicated zebrafish *ppp1r14ba* (chromosome 21) and *pppr14bb* (chromosome 7) genes show conserved synteny of these genes with gar and with cavefish. Paralog pairs were found for the flanking genes *slc8a4a*, *irfn4b,* and *pcxb* (Figure 4). Thus, these data are consistent with the four *ppp1r14a* and *ppp1r14b* genes, being the product of the teleost WGD. 

We next undertook a phylogenetic analysis of the *ppp1r14a* and *ppp1r14b* genes in fish. Trees were generated in IQ-TREE using the maximum likelihood method, and were re-rooted with *Lepisosteus oculatus* as the outgroup using FigTree. The duplicated *ppp1r14a* genes group clearly into distinct well conserved clusters (Figure S1). Corresponding homologs were found in clades that largely correlated with phylogenetic expectation. Zebrafish *ppp1r14aa* and *ppp1r14ab* each clustered near genes from other cypriniforms, such as *Cyprinus carpio* and *Astyanax mexicanus*. Many teleost species retained two *ppp1r14a* paralogs; however, several species only had a single homolog. For example, *Orysias latipes* (medaka, another popular model teleost) has only a single homolog that clusters with *ppp1r14aa* homologs, which is consistent with the loss of *ppp1r14ab* in the evolution of that lineage. Several salmonid species have additional paralogs. For example, *Oncorhynchus kisutch* (Coho salmon), *Oncorhynchus mykiss* (Rainbow trout/steelhead), and *Salmo salar* (Atlantic salmon) each have a *ppp1r14aa1* and a *ppp1r14aa2* which cluster with *Esox lucius* (Northern pike) *ppp1r14aa*. This is highly consistent with an additional salmonid specific WGD [8]. Overall, the phylogenetic analysis of *ppp1r14a* is consistent with a teleost WGD producing paralogs, which are retained in many lineages, but lost in others. 

The phylogenetic analysis of *ppp1r14b* genes showed a similar pattern to *ppp1r14a,* but fewer fish species retained both paralogs. Two large clusters formed for *ppp1r14ba* and *ppp1r14bb* (Figure S2). The clade for *ppp1r14ba* included several cypriniform genes, as well as genes from salmonids and the Atlantic herring (*Clupea harengus*)*,* each of which retained *ppp1r14bb* genes as well. Asian arowana (*Scleropages formosus*) *ppp1r14b* clustered with the *ppp1r14bb* clade, but no arowana *ppp1r14bb* was found. Additional salmonid duplications were apparent within both the *ppp1r14bb* and *ppp1r14ba* clades. Most other teleost lineages retained only a *ppp1r14bb* gene. Overall, the phylogenetic analysis is consistent with teleost WGD and loss of *ppp1r14ba* from many teleost lineages, however two paralogs in some lineages were retained, such as cypriniforms and salmonids, with additional duplication in salmonids. 

### 2.3. Expression Patterns of Cpi-17 and Phi-1 Paralogs During Early Zebrafish Development 

**Figure 5:**
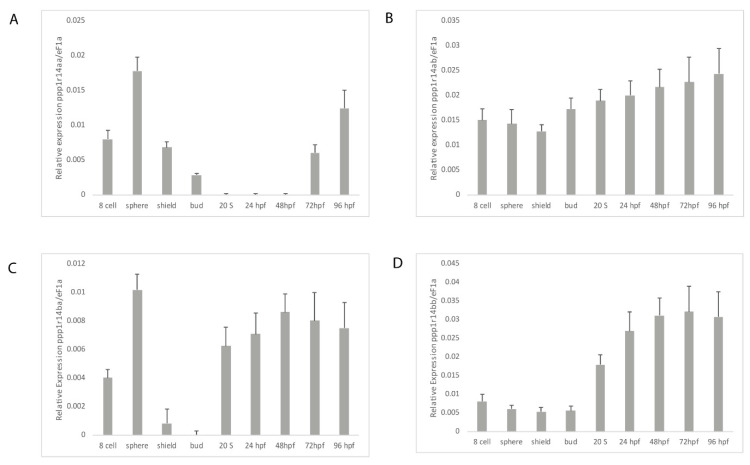
Expression of *ppp1r14a* and *ppp1r14b* paralogs during early embryonic development in zebrafish. Embryos were collected shortly after fertilization (8 cell), at sphere stage (4 hpf), shield stage (6 hpf), bud stage (10 hpf), 20 somite stage, Prim-5 stage (24 hpf), long-pec stage (48 hpf), protruding-month stage (72 hpf), and day 4 (96 hpf). Amplification of *eF1a* and total RNA without addition of reverse transcriptase were used as positive and negative controls, respectively. (A) Gene specific primers were used to detect *ppp1r14aa* (**A**), *ppp1r14ab* (**B**), *ppp1r14ba* (**C**), or *ppp1r14bb* (**D**). Values are means of three biological replicates performed in duplicate. Error bars represent standard deviation. All values are reported as expression relative to *ef1a*.

In order to determine if the duplicated *ppp1r14a* and *ppp1r14b* paralogs have diverged in their gene expression patterns, we utilized qRT-PCR. Wild type zebrafish embryos were collected at a variety of stages spanning early zebrafish development, including 8 cell stage (1.25 hpf) when gene expression is dominated by maternally deposited mRNA, sphere stage (4 hpf) when the mid blastula transition occurs and zygotic expression increases, shield stage (6 hpf) where gastrulation begins, bud stage (10 hpf) when germ layer formation finalizes, the 20 somite stage in late somitogenesis (19 hpf), 24 hpf, 48 hpf, 72 hpf, and 96 hpf. Gene specific primers (Table S2) were used to amplify *ppp1r14aa*, *ppp1r14ab*, *ppp1r14ba,* and *ppp1r14bb*. The expression of each gene was reported relative to the housekeeping gene *ef1a* [54]. Expression of *ppp1r14aa* was detected in maternally deposited mRNA, increased at sphere stage, then declined throughout gastrulation, becoming undetectable by late somitogenesis, and appeared again at 72 hpf (Figure 5A). Expression of *ppp1r14ab* was present maternally and zygotically, and showed no statistically significant change in expression during early development (Figure 5B). Maternal expression of *ppp1r14ba* was detected, increased at sphere stage, and then declined by gastrulation (Figure 5C). Expression was detected again by late somitogenesis, and remained high through the rest of early development (Figure 5C). Expression of *ppp1r14bb* was low from the 8 cell stage through the end of gastrulation, then increased during somitogenesis, and remained elevated for the rest of early development (Figure 5D).

The spatial gene expression patterns of individual *ppp1r14* family members have been characterized previously, and are consistent with our qRT-PCR results (Figure 5). The expression of *ppp1r14aa* was primarily detected in the smooth muscle of the developing gut and swim bladder [47], as well as spinal cord and hindbrain oligodendrocytes [48]. In contrast, *ppp1r14ab* is expressed primarily in the developing nervous system [50]. During early somitogenesis, it is expressed in the hindbrain neural plate and ventral mesoderm. By the 10–13 somite stage, it is highly enriched in rhombomere 4 and remains expressed in the cranial ganglion and diencephalon through day 5 [50]. *Ppp1r14ba* was found widely expressed in the central nervous system, retina and olfactory organ in 24 through 48 hpf embryos [49]. In contrast, *ppp1r14bb* was found primarily in somites during late somitogenesis, with additional expression in the head mesenchyme [49]. In 48 hpf embryos, *ppp1r14bb* was also expressed in pectoral fin musculature [49]. 

### 2.4. Inhibition of the Myosin Phosphatase by Cpi-17 Family Members

**Figure 6:**
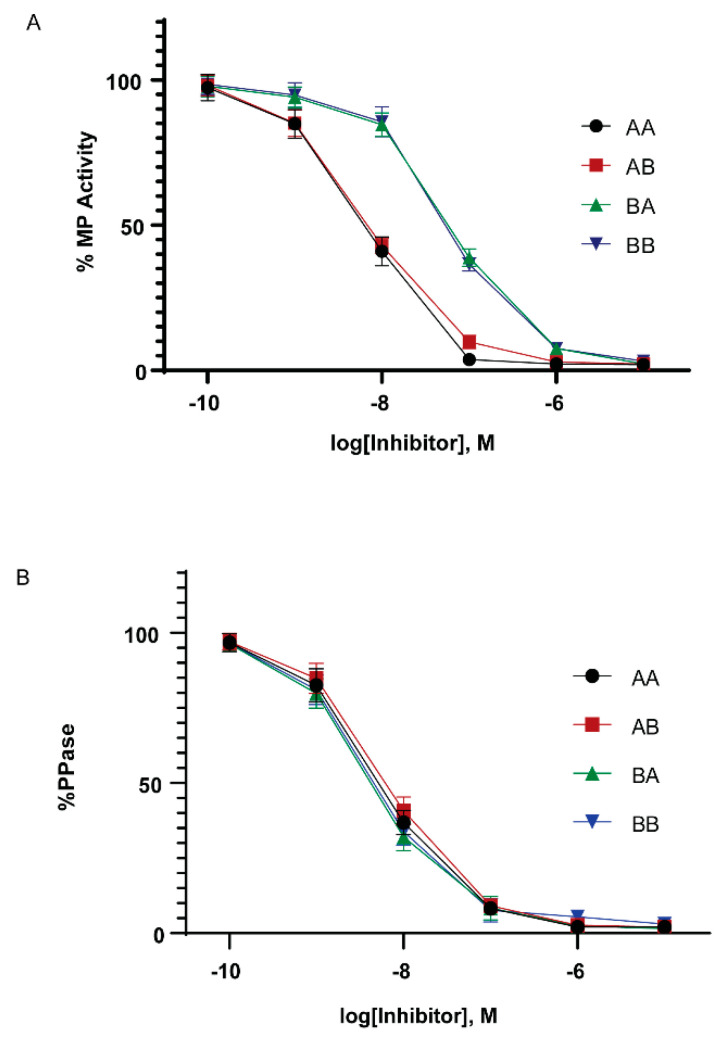
In vitro inhibition of myosin phosphatase by Cpi-17 and Phi-1 paralogs. (**A**) The myosin phosphatase holoenzyme was isolated from HEK cells and used to dephosphorylate recombinant Mlc2. Increasing concentrations of thiophosphorylated *ppp1r14aa* (AA, circle), *ppp1r14ab* (AB, red square), *ppp1r14ba* (BA, green triangle), or *ppp1r14bb* (BB, blue triangle) proteins were added, and the extent of Mlc2 dephosphorylation is reported as percent phosphatase activity. (**B**) Isolated PP1catalytic subunit was used to dephosphorylate Mlc2. Increasing concentrations of thiophosphorylated *ppp1r14aa* (AA, circle), *ppp1r14ab* (AB, red square), *ppp1r14ba* (BA, green triangle), or *ppp1r14bb* (BB, blue triangle) proteins were added, and the extent of Mlc2 dephosphorylation is reported as percent phosphatase activity. Each experiment was repeated three times in duplicate, and error bars indicate standard error.

In mammals, the ability to inhibit PP1 holoenzyme complexes is a hallmark of the Cpi-17 family. We therefore examined if the Cpi-17 and Phi-1 paralogs have undergone sub-specialization at the functional level. To test this, we relied on in vitro phosphatase assays and heterologous cellular systems. The coding regions for *ppp1r14aa*, *ppp1r14ab*, *ppp1r14ba,* and *ppp1r14bb* were subcloned into the bacterial expression vector pQE80. His-tagged recombinant proteins were expressed and purified, and the isolated proteins were thiophosphorylated. These proteins were then tested for their ability to inhibit the dephosphorylation of myosin light chain, by either purified PP1 catalytic subunit or purified myosin phosphatase holoenzyme (Mypt1 and PP1). We observed that all four proteins, when thiophosphorylated, were capable of inhibiting the purified PP1 catalytic subunit (Figure 6B), with IC50s of 5.5 +/- 1.4 nM (Cpi-17a), 6.5 +/- 1.9 nM (Cpi-17b), 4.3 +/- 1.8 nM (Phi-1a), 4.5 +/-1.7 nM (Phi-1b) and no statistically significant difference among the four proteins. Unphosphorylated proteins had no effect on phosphatase activity (data not shown). In contrast, we observed that Cpi-17a and Cpi-17b could inhibit the myosin phosphatase holoenzyme with IC50s of 6.9 +/- 1.6 nM and 6.9 +/- 1.5 nM, while Phi-1a and Phi-1b could inhibit the myosin phosphatase with IC50s of 64 +/- 12.7 nM and 58 +/- 13.1 nM (Figure 6A). In mammals, while both Phi-1 and Cpi-17 can inhibit the myosin phosphatase in vitro, Cpi-17 is 30–50 times more potent [31]. These data indicate that the greater affinity of Cpi-17 for the myosin phosphatase is conserved in zebrafish. In addition, we observed no significant difference in PP1-inhibition between Cpi-17a and Cpi-17b, or between Phi-1a and Phi-1b, indicating that the protein products of the paralogs have not functionally diverged significantly. 

**Figure 7:**
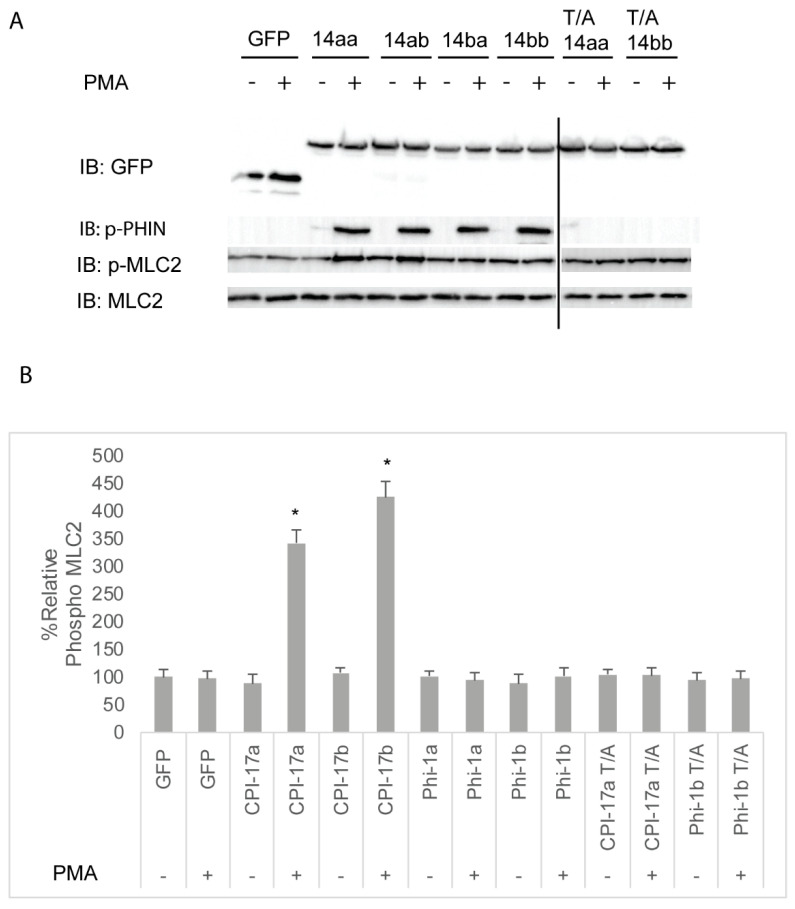
Differential inhibition of the myosin phosphatase by Cpi-17 family members in HeLa cells. (**A**) HeLa cells were transfected with either GFP alone, *ppp1r14aa, ppp1r14ab, ppp1r14ba, ppp1r14bb*, mutant *ppp1r14aa* lacking the regulatory phosphorylation site, or mutant *ppp1r14bb* lacking the regulatory phosphorylation site. Moreover, 15 h post transfection cells were treated with either 1 µM PMA or 0.1% DMSO for 3 h, and lysates were blotted with anti-GFP, anti-phospho-PHIN, anti-Mlc2, and anti phospho-Mlc2. (**B**) Mlc2 phosphorylation was normalized to total Mlc2 content, and is shown as fold change compared with cells expressing the empty GFP vector. Error bars indicate standard error, and an * indicates a statistically significant difference from control. Statistical significance was calculated using a one-factor ANOVA with Holm-Šídák post-hoc analysis, and is defined as *p* < 0.05 from at least 4 biological replicates.

We next sought to assess the ability of Cpi-17 family members to regulate myosin phosphorylation in a cellular system. HeLa cells were chosen, because they are highly sensitive to both increases and decreases in Mlc2 phosphorylation, and these changes can be detected using phospho-specific antibodies and the analysis of the actin cytoskeleton. In addition, HeLa cells do not express Cpi-17, and activation of PKC does not lead to increased myosin light chain phosphorylation. However, when functional Cpi-17 is expressed in HeLa cells, activating PKC results in myosin phosphatase inhibition, resulting in increased Mlc2 phosphorylation and a rearrangement of the actin cytoskeleton, including increase stress fiber formation [55]. Thus, HeLa cells were transfected with plasmids encoding GFP, GFP-Cpi-17a, GFP-Cpi-17b, GFP-Phi-1a, GFP-Phi-1b, GFP-Cpi-17a T31A, or GFP-Phi-1b T38A, and then either treated with either 1 µM of the PKC agonist PMA or 0.1% DMSO for 3 h. In the absence of PMA, phosphorylation of Cpi-17 or Phi-1 was barely detectible, but increased considerably after PMA treatment (Figure 7A). In the absence of PMA, none of the transfections resulted in any appreciable increase in Mlc2 phosphorylation (Figure 7B). In contrast, in the presence of PMA, Cpi-17a and Cpi-17b significantly increased Mlc2 phosphorylation. Importantly, if the conserved phosphorylation sites are mutated to alanine, Cpi-17a cannot be phosphorylated, and loses its ability to increase Mlc2 phosphorylation (Figure 7A,B).

We next determined if the increased Mlc2 phosphorylation resulted in changes in the actin cytoskeleton. HeLa cells normally have parallel stress fibers. However, the hyperphosphorylation of Mlc2 (for example, induced by overexpressing Mlc2 kinases) results in both an increase in the total number of stress fibers and the number of focused stress fibers radiating from a central point. As expected from the results in Figure 7, the expression of GFP, GFP-tagged Cpi-17, or GFP-Phi paralogs had no effect on stress fiber formation or orientation in the absence of PMA (Figure S3 and quantified in Figure S6). In the presence of PMA, expression of either Cpi-17a (Figure S3G,H) or Cpi-17b (Figure S3K,L) resulted in increased and focused stress fibers, consistent with the increased Mlc2 phosphorylation observed in Figure 7. Neither Phi-1a (Figure S3O,P) or Phi-1b (Figure S3S,T) resulted in any appreciable change in stress fiber phenotype. In addition, the phosphorylation site of CPI-17a is required for its ability to regulate the actin cytoskeleton (Figure S3W,X). 

Taken together, these experiments indicate that Cpi-17 is a more potent inhibitor of myosin phosphatase holoenzymes than Phi-1 in vitro. In cells, the overexpression of either paralog of Cpi-17, when phosphorylated, can result in a significant increase in Mlc2 phosphorylation and a dramatic rearrangement of the actin cytoskeleton. Overexpression of either paralog of Phi-1, in contrast, whether phosphorylated or not, did not appreciably alter Mlc2 phosphorylation or actin stress fiber formation. The extent to which Phi-1 regulates Mlc2 phosphorylation in other organisms is controversial. While phosphorylated Phi-1 causes increased actomyosin contraction in cultured smooth muscle strips [28], the injection of Phi-1 does not restore PKC-induced muscle contraction in Cpi-17-null avian smooth muscle [56], and Phi-1 knockdown in HeLa cells does not alter Mlc2 phosphorylation [57]. Our observations agree with previous research that shows that Phi-1 is a poor myosin phosphatase inhibitor and likely functions by regulating distinct PP1 holoenzymes.

### 2.5. The Highly Conserved PHIN Domain of Cpi-17 is Responsible for High Affinity Myosin Phosphatase Inhibition

**Figure 8:**
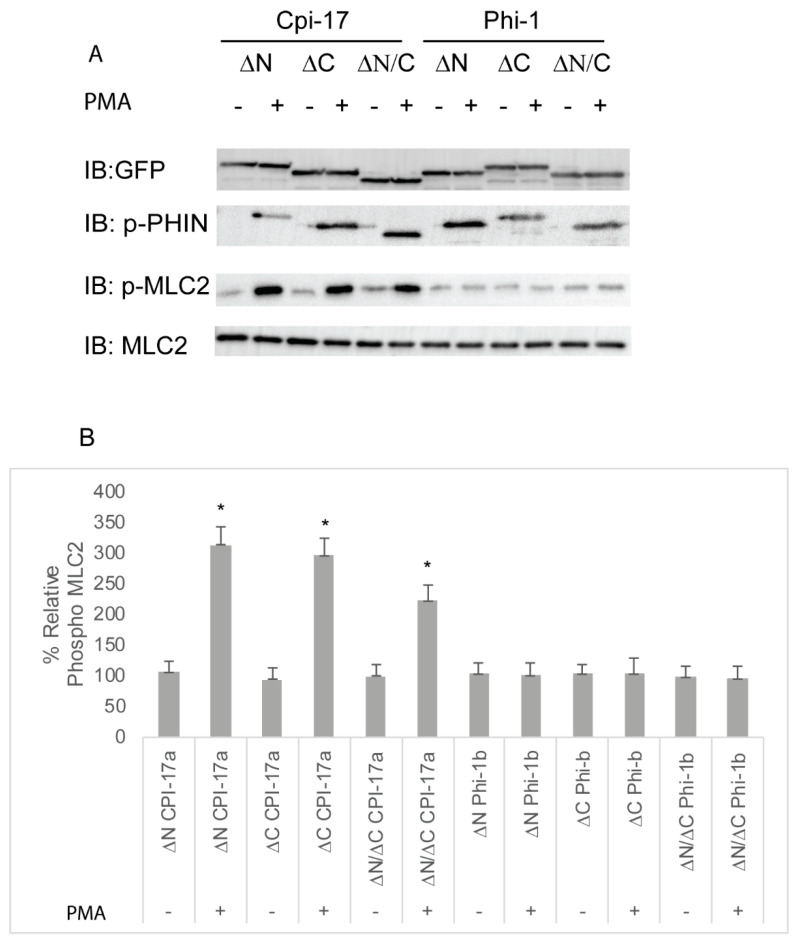
Truncation mutants of Cpi-17 family members highlight the critical role of the PHIN domain in myosin phosphatase inhibition. (**A**) HeLa cells were transfected with either *ppp1r14aa* lacking the N-terminus (ΔN), *ppp1r14aa* lacking the C terminus (ΔC), *ppp1r14aa* lacking both the N and C termini (ΔN/ΔC)*, ppp1r14bb* lacking the N-terminus (ΔN), *ppp1r14bb* lacking the C-terminus (ΔC), or *ppp1r14bb* lacking both the N- and C-termini (ΔN/ΔC). Notably, 15 h post transfection cells were treated with either 1 µM PMA or 0.1% DMSO for 3 h, and lysates were blotted with anti-GFP, anti-phospho-PHN, anti-Mlc2, or anti phospho-Mlc2. (**B**) Mlc2 phosphorylation was normalized to total Mlc2 content, and is shown as fold change compared with cells expressing the empty GFP-vector. Error bars indicate standard error, and an * indicates a statistically significant difference from control. Statistical significance was calculated using a one-factor ANOVA with Holm–Šídák post-hoc analysis and is defined as *p* < 0.05 from at least four biological replicates.

The amino acids sequences of Cpi-17a and Phi-1b are conserved within the central 89 amino acid PHIN domain (49% identity and 72% similarity), but each protein has a unique N- and C-terminus. To determine which amino acid sequences were responsible for the differential potency of Cpi-17 and Phi-1, we generated deletion constructs in both Cpi-17a and Phi1b, removing only the N-terminal loop (ΔN), the C-terminal loop (ΔC), or both, leaving just the PHIN domain (ΔN/ΔC). These constructs were then expressed in HeLa cells, and either treated with control DMSO or PMA to induce phosphorylation. None of the plasmids resulted in increased phosphorylation in the absence of PMA (Figure 8). In the presence of PMA, deletion constructs of CPI-17a lacking either the N or C terminus strongly induced Mlc2 phosphorylation, and the expression of the PHIN domain alone (ΔN/ΔC) was sufficient to induce Mlc2 phosphorylation (Figure 8). All Phi-1 deletions were phosphorylated in the presence of PMA, but had no effect on Mlc2 phosphorylation (Figure 8). These results were then confirmed by analyzing actin cytoskeletal rearrangements (Figure S4 and quantified in Figure S6). Again, all three Cpi-17 deletions resulted in a noticeable increase in actin stress fiber formation in the presence of PMA (Figure S4). None of the Phi-1 deletions induced actin stress fibers, and none of the constructs altered the actin cytoskeleton in the absence of PMA (Figure S4). 

**Figure 9:**
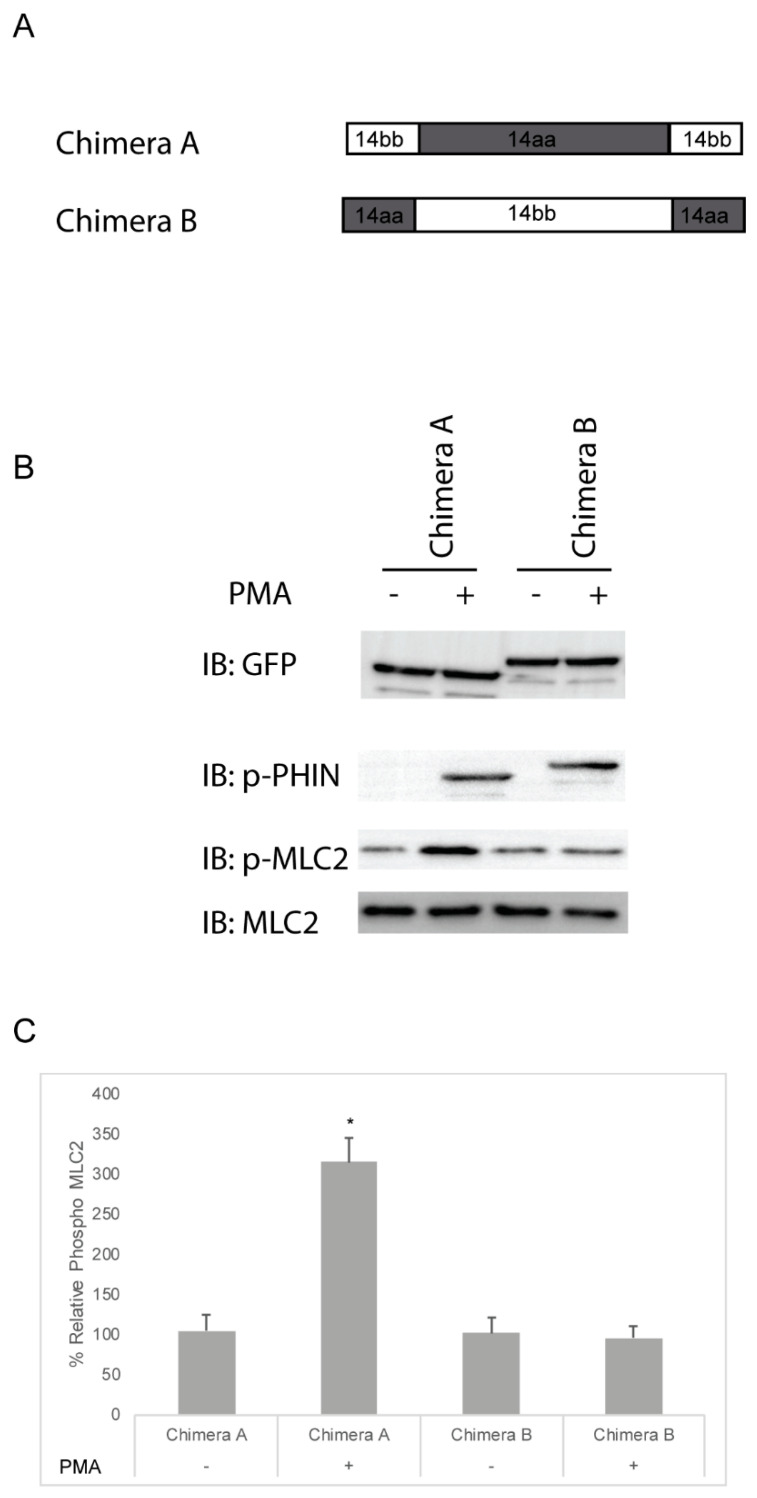
Cpi-17/Phi-1 chimeric proteins indicate that the PHIN domain is required for Myosin Phosphatase specificity. (**A**,**B**) HeLa cells were transfected with either chimera A, which contains the PHIN domain of *ppp1r14aa* and the N and C-termini of *ppp1r14bb*, or chimera B, which contains the PHIN domain of *ppp1r14bb* and the N and C-termini of *ppp1r14aa*. Notably, 15 h post transfection cells were treated with either 1 µM PMA or 0.1% DMSO for 3 h, and lysates were blotted with anti-GFP, anti-phospho-PHIN, anti-Mlc2, and anti phospho-Mlc2. (**C**) Mlc2 phosphorylation was normalized to total Mlc2 content and is shown as fold change compared with cells expressing the empty GFP-vector. Error bars indicate standard error, and an * indicates a statistically significant difference from control. Statistical significance was calculated using a one-factor ANOVA with Holm–Šídák post-hoc analysis and is defined as *p* < 0.05 from at least four biological replicates.

The deletion experiments were then extended with the use of chimeric constructs. Chimera A was constructed with the PHIN domain of Cpi-17 and the N- and C-termini of Phi-1, while Chimera B contained the PHIN domain of Phi-1 and the N- and C-termini of Cpi-17 (Figure 9A). Both Chimera A and Chimera B were phosphorylated in the presence of PMA (Figure 9B), but only Chimera A increased Mlc2 phosphorylation (Figure 9B and quantified Figure 9C). The analysis of stress fiber formation mirrored the analysis of Mlc2 phosphorylation, with Chimera A causing increased stress fiber formation in the presence of PMA, and Chimera B having no impact on stress fiber formation (Figure S5 and quantified Figure S6). Through the deletion and chimeric analysis, we have found that the PHIN domain of Cpi-17 is necessary and sufficient for the specific inhibition of the myosin phosphatase. This also indicates that the conserved mechanistic differences between Phi-1 and Cpi-17 are encoded by differences in the conserved PHIN domains, and not the divergent N- and C-termini. 

### 2.6. Expression of Cpi-17 Paralogs, But Not Phi-1 Paralogs, Rescue Knockdown of ppp1r14ab in Zebrafish Embryos

**Figure 10:**
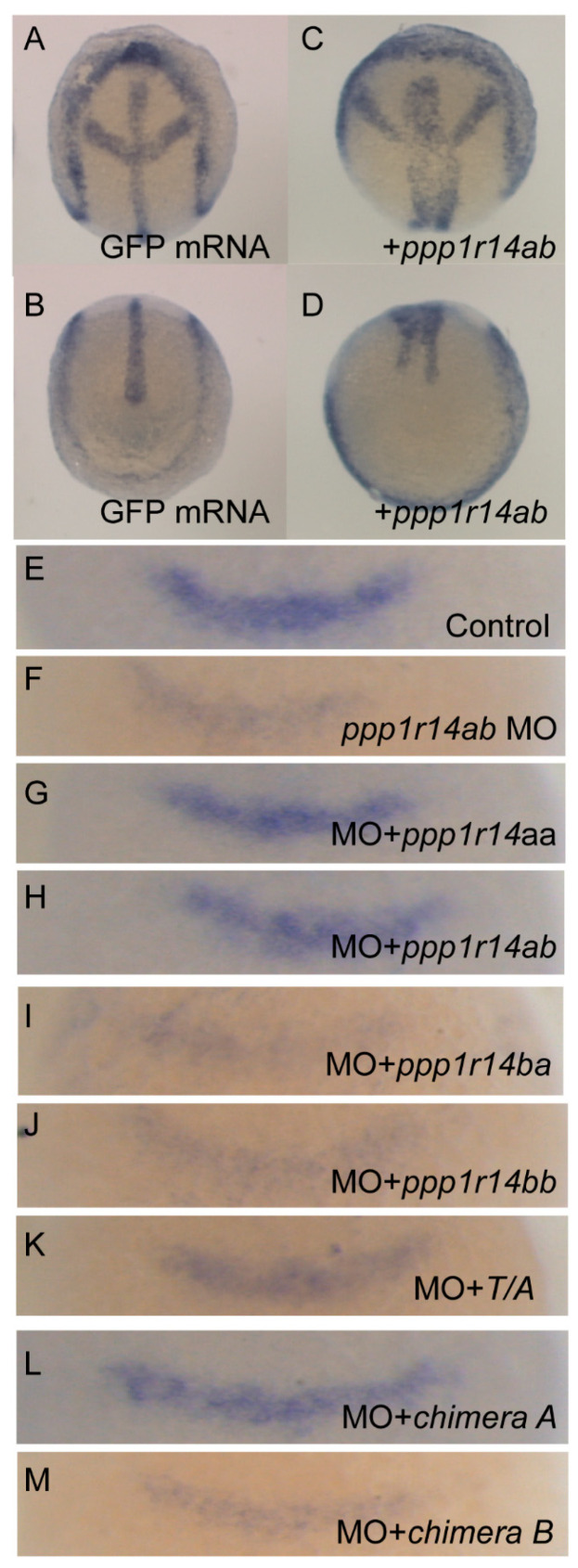
Expression of Cpi-17 paralogs, but not Phi-1 paralogs, can rescue *ppp1r14ab* loss-of-function. Embryos at bud stage stained with *hgg1* (prechordal plate), *shh* (midline), *pax2.1* (midbrain-hindbrain boundary), and *dlx3* (neural plate) after injection of 200 pg GFP (**A**,**B**) or 200 pg *ppp1r14aa* mRNA (**C**,**D**). (**E**–**M**) embryos were collected at 10.5 hpf and stained with *fgf3* after injection of 4ng of control MO (**E**), 4 ng of *ppp1r14ab* MO (**F**), 50pg of *ppp1r14aa* mRNA + 4 ng of *ppp1r14ab* MO (**G**), 50pg of *ppp1r14ab* mRNA + 4 ng of *ppp1r14ab* MO (**H**), 50pg of *ppp1r14bb* mRNA + 4 ng of *ppp1r14ab* MO (**I**), 50pg of *ppp1r14aa* mRNA + 4 ng of *ppp1r14ab* MO (**J**), 50pg of *ppp1r14aa* T38A mRNA + 4 ng of *ppp1r14ab* MO (**K**), 50 pg chimera A mRNA + 4 ng of *ppp1r14ab* MO (**L**), or 50ng chimera B mRNA 4 ng of ppp1r14ab MO (**M**).

PP1 has many critical functions in early development, including regulation of actomyosin contractility through myosin phosphatase, as well as multiple myosin-independent functions. Inhibition of PP1 results in severe defects in morphogenetic cell movements and patterning. Mis-regulation of PP1 results in severe gastrulation defects inducing characteristic phenotypes, including a shortened and broadened body axis, reduced axial migration of the prechordal plate, disruption of cell polarity and changes in cellular protrusive activity [58,59]. We anticipated that the overexpression of active phosphatase inhibitors, such as Cpi-17 or Phi-1, would disrupt early development. To test the effect of overexpression of Cpi-17 family members on cell movements during gastrulation, one-cell stage zebrafish embryos were injected with increasing doses (25,50,100, or 200 pg) mRNA encoding *ppp1r14aa*, *ppp1r14ab*, *ppp1r14ba*, *ppp1r14bb*, Chimera A, or Chimera B. Additional sets of embryos were injected with 200 pg mRNA encoding GFP (negative control) or unphosphorylatable mutants of *ppp1r14aa* (T/A) or *ppp1r14bb* (T/A). The embryos were then grown to the end of germ layer formation (bud stage), and body axis elongation was assayed by staining with a cocktail of four in situ probes (*shh* to mark the notochord, *hgg1* to mark the prechordal plate, *pax2.1* to mark the midbrain hindbrain boundary (also called *pax2a*), and *dlx3* to mark the neural plate). GFP-injected embryos were indistinguishable from uninjected embryos (Figure 10A,B), while injection of *ppp1r14ab* mRNA resulted in a dramatically broader notochord and presomitic mesoderm (Figure 10C,D), consistent with reduced convergence and extension movements during gastrulation. We observed a dose-dependent gastrulation defect with the injection of *ppp1r14aa*, *ppp1r14ab*, *ppp1r14ba*, *ppp1r14bb,* Chimera A, or Chimera B (Figure S7), which is consistent with prior observations using *ppp1r14aa* and *ppp1r14bb* [36]. In contrast, injection of 200 pg mRNA encoding the unphosphorylatable mutants of *ppp1r14aa* or *ppp1r14bb* did not induce any observable gastrulation defect (Figure S7). Overall, 50 pg was the highest dose that consistently failed to produce a gastrulation defect and was chosen for the rescue experiments in Figure 10E–M. This provided direct evidence that all orthologs and both chimeras are active if overexpressed in zebrafish, and that the phosphorylation of the PHIN domain is required for in vivo function. 

The only well characterized loss-of-function model for a Cpi-17 family member in zebrafish is *ppp1r14ab*, which was identified as a *hoxb1* target that regulates expression of *fgf3* in rhombomere 4 during hindbrain development [50]. We recapitulated the prior observation that morpholino knockdown of *ppp1r14ab* results in reduced expression of *fgf3* in rhombomere 4 (Figure 10E,F), and that co-injection of *ppp1r14ab* mRNA rescues the morphant phenotype (Figure 10H) [50]. Co-injection of mRNA for *ppp1r14aa* rescued knockdown of *ppp1r14ab* (Figure 10G), consistent with our in vitro evidence that *ppp1r14aa* and *ppp1r14ab* are functionally equivalent. In contrast, neither *ppp1r14ba* nor *ppp1r14bb* could rescue knockdown of *ppp1r14ab*, further demonstrating that the Phi-1 paralogs are genetically distinct from Cpi-17 (Figure 10I,J). Mutating the inhibitory phosphorylation site of *ppp1r14aa* rendered it unable to rescue knockdown of *ppp1r14ab* (Figure 10K), confirming that in vivo function of Cpi-17 protein requires phosphorylation. Finally, Chimera A, but not Chimera B, mRNA could rescue *ppp1r14ab* knockdown, indicating that the PHIN domain is responsible for the biochemical and physiological differences between Cpi-17 and Phi-1.

## 3. Materials and Methods 

### 3.1. Animal Care

Wild-type zebrafish (*Danio rerio*) embryos (AB or TU) were obtained through natural spawning, and were maintained and staged as described previously [60]. All experiments were approved by and conducted in accordance with the guidelines established by the Institutional Animal Care and Use Committee (IACUC) at the University of the Pacific. The approved IACUC protocols were as follows: 13R02 (31/10/13), 16R10 (31/10/16) and 19R01 (31/10/19). 

### 3.2. Cell Culture

HeLa and HEK293T cells (obtained from the ATCC, Manassas, VA, USA) were maintained in Dulbecco’s modified Eagle medium (DMEM), containing 25 mM D-glucose and 1 mM sodium pyruvate supplemented with 10% fetal bovine serum (Thermo Fisher Scientific, Waltham, MA, USA). All cells were maintained in a 5% CO_2_ incubator at 37 °C. Transfections were performed using Lipofectamine LTX reagent (Thermo Fisher Scientific, Waltham, MA, USA) using the manufacturer’s instructions. Cells were plated in 6-well petri dishes on coverslips coated with 1 μg of fibronectin (Sigma-Aldrich F1141, St Louis, MO, USA). 

### 3.3. Cloning ppp1r14 Family Members and Other Plasmids

To generate full-length GFP-fusion plasmids, the coding regions of *ppp1r14aa*, *ppp1r14ab*, *ppp1r14ba*, and *ppp1r14bb* were amplified from total cDNA of 24 hpf zebrafish embryos, using the primers in (Table S2), digested with EcoRI and SalI and ligated into pEGFP-C1 (*ppp1r14aa*) or pEGFP-C2 (*ppp1r14ab, ba, bb*), digested with EcoRI and SalI. The conserved phosphorylation sites in *ppp1r14aa* (T31) and *ppp1r14bb* (T38) were mutated using the primers in Table S2 and the QuikChange (Agilent, Santa Clara, CA, USA) site-directed mutagenesis kit. Truncation mutants, Chimera A, and Chimera B were generated using primers in Table S2 and ligated into pEGFP-C1.

To generate full-length his-tag fusion plasmids, each ortholog was amplified using the primers in Table S2, digested with BamHI and SalI (*ppp1r14ab, ba, bb*) or BglII and SalI (*ppp1r14aa*), and ligated into pQE80 digested with BamHI and SalI. To generate plasmids for mRNA synthesis, *ppp1r14aa* and *ppp1r14bb* were amplified using primers in Table S2, digested with ClaI and EcoRI, and ligated into pCS2+ digested with ClaI and EcoRI. In addition, EcoRI-SalI fragments of *ppp1r14ab*, *ppp1r14ba, ppp1r14aa-T31A*, *ppp1r14bb-T38A*, Chimera A, and Chimera B were subcloned into pCS2+ digested with EcoRI and XhoI. For in situ hybridization, the 5′ and 3′ UTR and coding regions of each gene were amplified using the primers in Table S2, digested with BamHI and ClaI (BlgII and ClaI for *ppp1r14aa*), and ligated into pCS2 digested with BamHI and ClaI. Plasmids for GST-Mlc2, myc-Mypt1 (1–300), GFP-tagged PP1, *hgg1, shh1, pax2.1* and *dlx3b* were described previously [58,60]. The plasmid for *fgf3* was the kind gift of Alex Nechiporuk (specification of epibranchial placodes in zebrafish) [61]. 

### 3.4. Morpholinos and Zebrafish Injection

A previously characterized morpholino directed against *ppp1r14ab* was obtained from Gene Tools 5′-CACCCGATTCGCAGCCATCTCCAGA-3′ [50]. To generate mRNA for each Cpi-17 gene family member, purified pCS2+ plasmid DNA with each gene inserted was digested with NotI, and mRNA was synthesized using the mMessage mMachine SP6 kit (Ambion, Austin, TX, USA). Zebrafish embryos were injected with the indicated concentration of morpholino or mRNA at the one-cell stage as described, with needles calibrated to inject 1 nL of fluid. Embryos were kept in E3 media at 28.5 °C until the desired stage.

### 3.5. In Situ Hybridization

Whole-mount in situ hybridization was performed using digoxigenin-labeled antisense RNA probes. Embryos and probes were processed as described previously [60].

### 3.6. Quantitative Reverse Transcriptase PCR

Trizol was used to isolate mRNA, and quantitative RT-PCR was performed using iTAQ SYBR Green I supermixes with RT (Biorad, Hercules, CA, USA). The experiments were run on a Biorad CFX96 instrument, and analyzed using CFX Maestro software (Biorad, Hercules, CA, USA) for 40 cycles, following manufacturer’s instructions. Expression levels of the tested genes were normalized to *ef1a* as described [54]. All assays were performed in duplicate in a 96 well format, and 3 biological replicates were analyzed. The expression ratios of each gene were obtained using the 2−ΔΔCt method [54].

### 3.7. Antibodies and Western Blots

Then, 15 h post transfection, cells were treated with either 1 µM PMA (a PKC agonist, Sigma-Aldrich, St Louis, MO, USA) or 0.1% DMSO for 3 h, then washed in PBS, and lysed in RIPA buffer. Cleared lysates were run on 12% SDS-PAGE and blotted with anti-GFP (Santa Cruz B2, Santa Cruz, CA, USA), anti-phospho-Cpi-17 (Santa Cruz-17560, Santa Cruz, CA, USA), anti-Mlc2 (Cell Signaling #3672, Danvers, MA, USA), and anti phospho-Mlc2 (Cell Signaling #3675, Danvers, MA, USA).

### 3.8. Stress Fiber Assays

HeLa cells were plated on fibronectin coated coverslips, followed by transfections, as described [62,63]. Approximately 15–18 h post transfections cells were fixed in 4% paraformaldehyde, permeabilized in 0.2% Triton-X, and washed with PBS. Cells were stained with Alexa 568 Phalloidin (Thermo Fisher Scientific, Waltham, MA, USA) and DAPI (Thermo Fisher Scientific, Waltham, MA, USA). Assaying for stress fibers was performed as described [63]. A minimum of 25 GFP-positive transfected HeLa cells were randomly selected per experimental condition. The cells were then qualitatively scored visually, using ImageJ and categorized by stress fiber phenotype. A normal stress fiber phenotype was defined as cells containing three or more stress fibers passing through the majority of the cytoplasm. Increased stress fiber phenotypes were scored when cells had aggregated brightly staining stress fibers, or had more numerous and focused stress fibers. All experiments were replicated 3–5 times with the total *n* in parenthesis, and the data are reported by percent. Slides were visualized using Leica DMIRE2 inverted fluorescence microscope using metamorph software (Molecular Devices, Sunnyvale, CA, USA), a Plan Apo 40×/0.85 NA objective and Yokogawa CSU-X1 spinning disc confocal with a QuantEM: 5125C camera (QuantEM, San Jose, CA, USA).

### 3.9. Phosphatase Assays

His-tagged recombinant *ppp1r14aa*, *14ab*, *14ba* and *14bb* were expressed in, and purified from, BL21 DE3 RPIL (Agilent, Santa Clara, CA, USA) *E. coli* using Nickle NTA agarose (Qiagen, Germantown, MD, USA) under native conditions, following manufacturer’s instructions. The purified proteins were thiophosphorylated with ATPγs, and recombinant PKC delta (Thermo Fisher Scientific, Waltham, MA, USA) in the presence of lipid activator, and phosphorylation was verified by Western blot analysis. GST-Mlc2 was purified and phosphorylated as described [58]. Active myosin phosphatase holoenzyme was purified as described [58]. Phosphorylated Mlc2 was incubated for 30 min at 30 °C, with either purified Myosin Phosphatase or recombinant PP1 (New England Biolabs, Ipswich, MA, USA) and various concentrations of inhibitors. Samples were then analyzed by phos-tag gel electrophoresis, and the ratio of phosphorylated and dephosphorylated MLC2 was calculated using Biorad Imagelab 4.1 (Biorad, Hercules, CA, USA) [64]. IC50s were calculated using GraphPad Prism8 (GraphPad Software, San Diego, CA, USA).

### 3.10. Homology and Phylogenetic Analysis

Genomicus (http://www.genomicus.biologie.ens.fr/genomicus) and Ensembl (https://www.ensembl.org) gene trees were used to identify *ppp1r14aa*, *ppp1r14ab*, *ppp1r14ba*, and *ppp1r14bb* homologs in various fish species (Table S1). In order to find additional fish homologs, the *Danio rerio* sequences were used as input in blastp of the Refseq database using default settings. The sequences in Table S1 were used in the phylogenetic analysis. Species with homologs containing large regions without homology were excluded from analysis. Protein sequence alignments were performed for all identified fish orthologs *ppp1r14aa*, *ppp1r14ab*, *ppp1r14ba*, and *ppp1r14bb* in CLC Sequence Viewer, using the slow (very accurate) algorithm, with default gap costs and end gap costs, as any other. The maximum likelihood method was used to generate trees in IQ-TREE, and bootstrap analysis was performed with 1000 replicates to determine the reliability of nodes. Trees were re-rooted with *Lepisosteus oculatus* (spotted gar) as the outgroup using FigTree v1.4.3 (http://tree.bio.ed.ac.uk/software/figtree/) A phylogenetic tree of the Cpi-17 family in *Danio rerio* and *Homo sapiens* was created using the PHIN domains. The PHIN domains were found visually using protein alignments. Genomicus genome browser was used to create synteny trees. 

### 3.11. Statistical Analysis

Statistical analysis was performed using MS Excel and Daniel’s XL Tool box (https://www.xltoolbox.net/). For quantitative data, mean and standard deviation are shown, and statistical significance was determined using a 1-factor ANOVA and Holm–Šídák post-hoc comparisons. For all tests, a *p* < 0.05 was considered statistically significant. 

## 4. Conclusions

We have identified four members of the Cpi-17 (*ppp1r14*) family of phosphatase inhibitors that are expressed in early zebrafish development. Two paralogs of Cpi-17 (*ppp1r14aa* and *ppp1r14ab*) appear to be the product of whole genome duplication, and are conserved in many fish species. The two *ppp1r14a* paralogs have distinct expression patterns, with *ppp1r14aa* expressed primarily in smooth muscle, while *ppp1r14ab* is primarily neural. Two paralogs of *ppp1r14b* also occur in zebrafish, the primarily neural expressed *ppp1r14ba* and the primarily skeletal muscle expressed *ppp1r14bb*. In vitro assays showed that all four proteins are capable of inhibiting purified PP1, but that both Phi-1a and Phi-1b had lower specificity for the myosin phosphatase holoenzyme. When tested in heterologous cell systems, the two Cpi-17 proteins could robustly increase Mlc2 phosphorylation by inhibiting myosin phosphatase, while the two Phi-1 proteins had no effect on Mlc2 phosphorylation. Using deletion and chimeric analyses, we identified that the central PHIN domain of Cpi-17 is responsible for myosin phosphatase inhibition. Finally, we demonstrated that either *ppp1r14aa* or *ppp1r14ab* mRNA expression could rescue the *ppp1r14ab* knockdown phenotype. In contrast, neither *ppp1r14ba* nor *ppp1r14bb* mRNA could rescue the *ppp1r14ab* knockdown phenotype. Therefore, we conclude that the Phi-1 paralogs are much weaker myosin phosphatase inhibitors than the Cpi-17 paralogs both in vitro and in vivo. Taken altogether, our data indicate that each pair of paralogs have retained similar biochemical specificity, but have diverged in gene expression pattern. 

## Supplementary Materials

The following are available online at https://www.mdpi.com/1422-0067/21/16/5709/s1, Figure S1. A ppp1r14a phylogenetic tree. Figure S2. A ppp1r14b phylogenetic tree. Figure S3. Phosphorylated Cpi-17a or Cpi-17b but not Phi-1a or Phi-1b increase actomyosin stress fiber formation in HeLa cells. Figure S4. Deletion analysis of Cpi-17 and Phi-1 indicates that the PHIN domain of Cpi-17 is sufficient to induce stress fiber formation in HeLa cells. Figure S5. Chimera A but not Chimera B is sufficient to induce stress fiber formation in HeLa cells. Figure S6. Quantification of stress fiber phenotypes. Figure S7. Overexpression of Cpi-17 family members in zebrafish results in gastrulation defects. Table S1. Sequences used in this study. Table S2. Primers used in this study.

## Abbreviations

PP1	Protein phosphatase 1
Mlc2	Myosin light chain 2
Mypt1	Myosin phosphatase targeting subunit 1
Cpi-17	C-kinase-activated PP1 inhibitor of 17kDa—*ppp1r14a*
Phi-1	PP1 holoenzyme inhibitor—*ppp1r14b*
KEPI	Kinase-C-enhanced PP1 inhibitor
GBPI	Gastro-intestinal and brain specific PP1 inhibitor
PHIN	PP1 holoenzyme inhibitory
CE	Convergent extension
qPCR	Quantitative polymerase chain reaction
MO	Morpholino antisense oligonucleotide
PKC	Protein kinase C
