# Supplementary material for: The Evolution of Duplicated Genes of the Cpi-17/Phi-1 (ppp1r14) Family of Protein Phosphatase 1 Inhibitors in Teleosts
Source: Int J Mol Sci. 2020 Aug 9;21(16):5709. doi: 10.3390/ijms21165709 (PMC7460850; doi:10.3390/ijms21165709)
Supplement: Supplementary file 1 [file ijms-21-05709-s001.pdf]

Figure S1. A *ppp1r14a* phylogenetic tree. Ppp1r14a maximum likelihood tree using fish homologs found by blastp is consistent with Genomicus and Ensembl gene trees and supports a whole genome duplication.

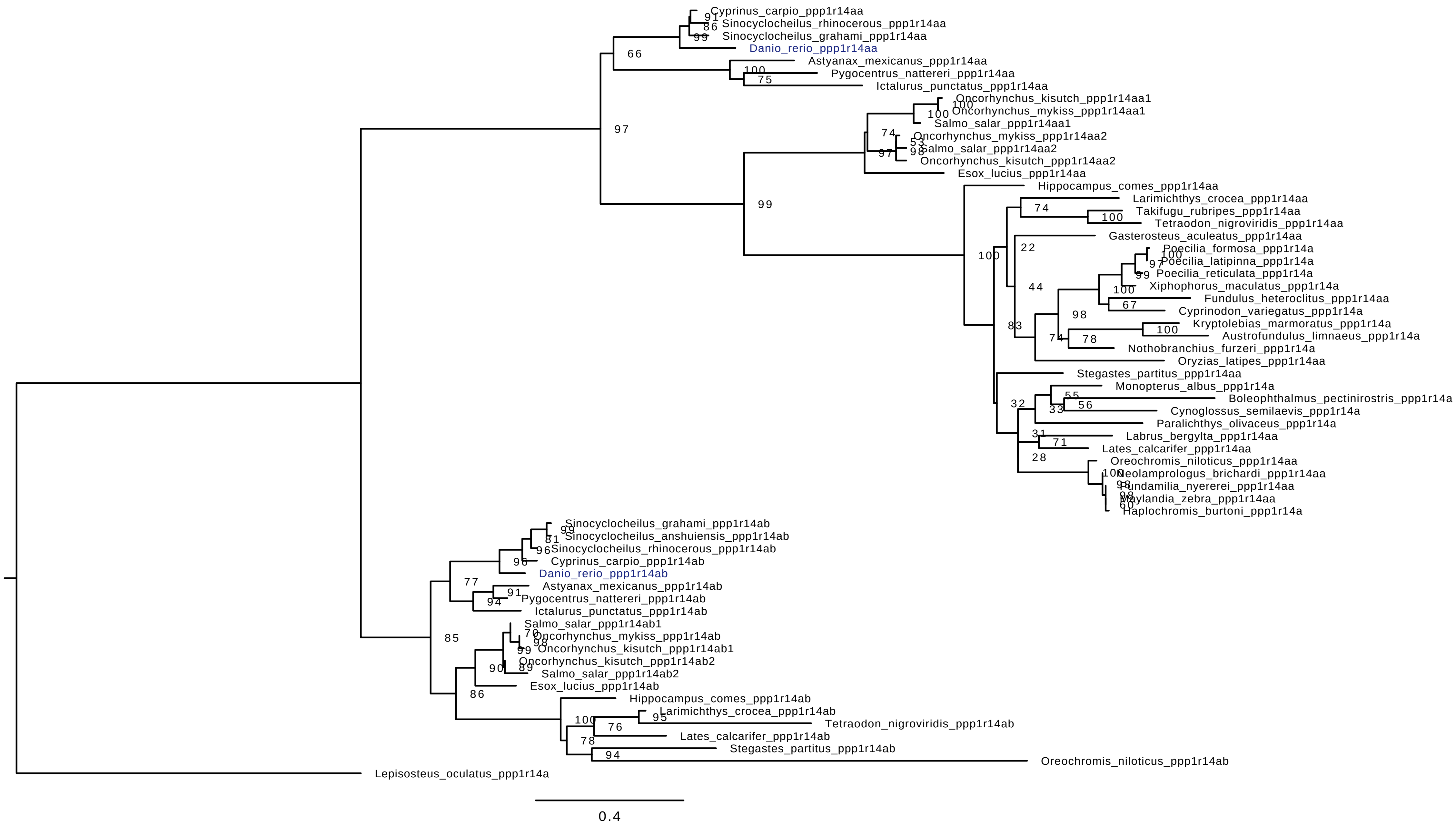

Figure S2. A *ppp1r14b* phylogenetic tree. Ppp1r14b maximum likelihood tree using fish homologs found by blastp indicates that the *ppp1r14b* homologs in *Danio rerio* are the result of a whole genome duplication.

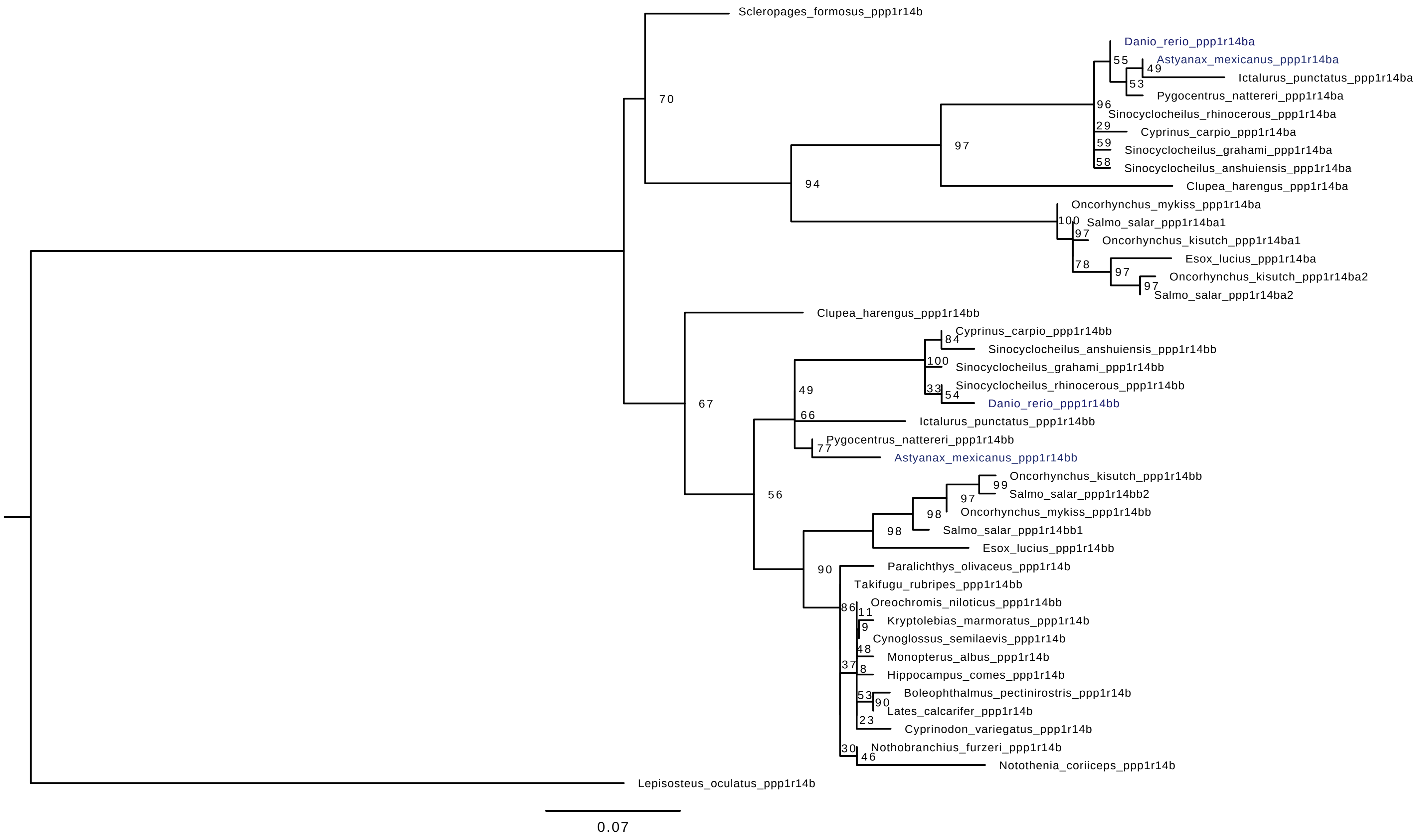

Figure S3. Phosphorylated Cpi-17a or Cpi-17b but not Phi-1a or Phi-1b increase actomyosin stress fiber formation in HeLa cells. HeLa cells were transfected with either GFP (A-D), GFP-Cpi-17a (E-H), GFP-Cpi-17b (I-L), GFP-Phi-1a (M-P), GFP-Phi-1b (Q-T), GFP-Cpi-17a T31A (U-X), or GFP-Phi-1b T38A (Y-BB). Cells were treated with either 0.1% DMSO (A, B, E, F, I, J, M, N, Q, R, U, V, Y, Z) or 1 µM PMA (C, D, G, H, K, L, O, P, S, T, W, Y, AA, BB) for 3 hours prior to fixation. All cells were fixed and stained with DAPI and Alexa 568-phalloidin to stain the nucleus and actin cytoskeleton, respectively, and imaged with confocal microscopy. Black and white images show phalloidin staining, while color images are a merge of DAPI (blue), GFP (green), and phalloidin (red). White bar indicates 20 µm. Representative cells are shown from a minimum of three biological replicates.

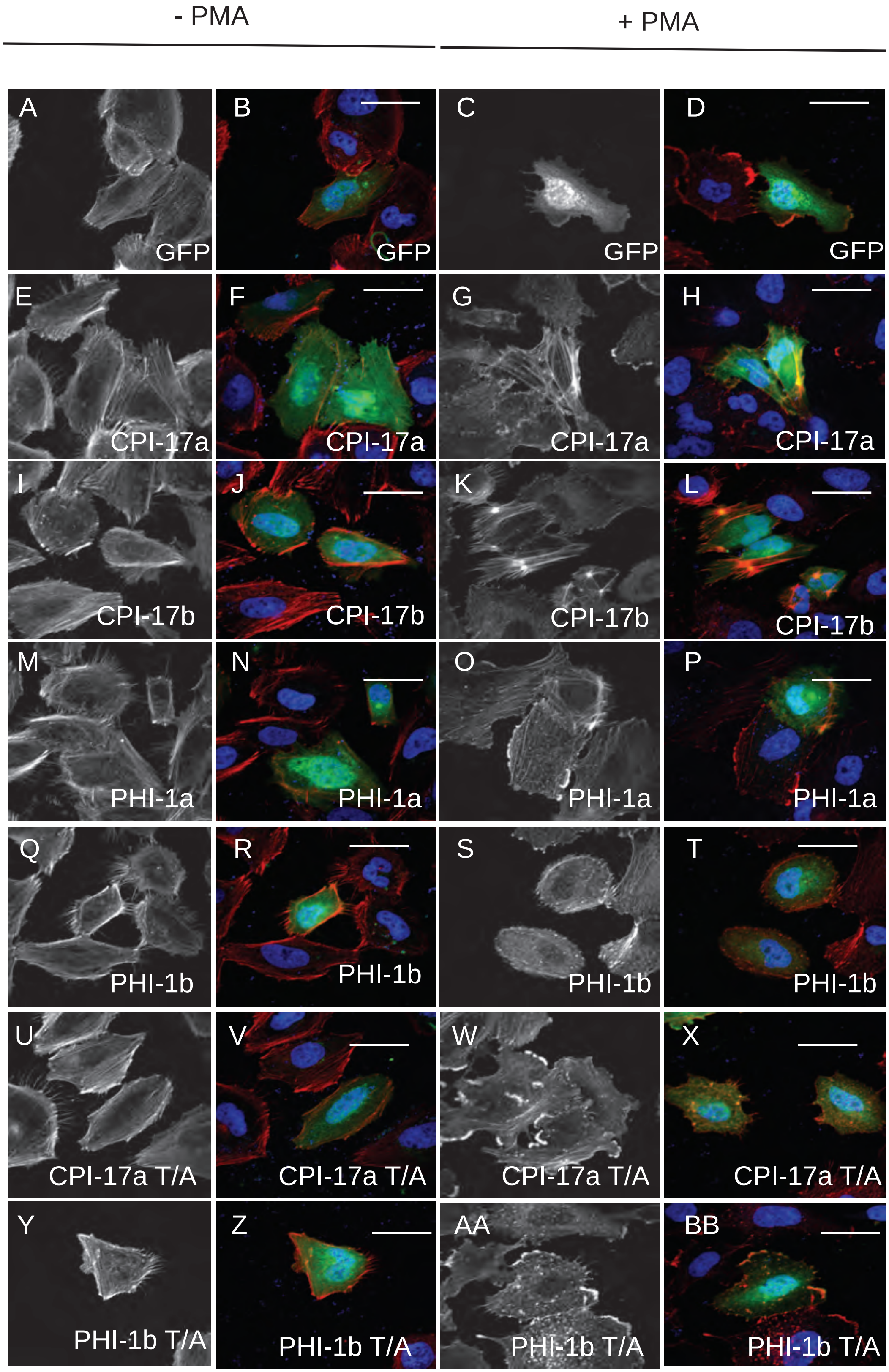

Figure S4. Deletion analysis of Cpi-17 and Phi-1 indicates that the PHIN domain of Cpi-17 is sufficient to induce stress fiber formation in HeLa cells. HeLa cells were transfected with either GFP-Cpi-17a ΔN (A-D), GFP-Cpi-17a ΔC (E-H), GFP-Cpi-17a ΔN/ΔC (I-L), GFP-Phi1b ΔN (M-P), GFP-Phi-1b ΔC (Q-T), or GFP-Phi1b ΔN/ΔC (U-BB). Cells were treated with either 0.1% DMSO (A, B, E, F, I, J, M, N, Q, R, U, V) or 1 μM PMA (C, D, G, H, K, L, O, P, S, T, W, X) for 3 hours prior to fixation. All cells were fixed and stained with DAPI and Alexa 568-phalloidin to stain the nucleus and actin cytoskeleton, respectively, and imaged with confocal microscopy. Black and white images show phalloidin staining, while color images are a merge of DAPI (blue), GFP (green), and phalloidin (red). White bar indicates 20 μm. Representative cells are shown from a minimum of three biological replicates.

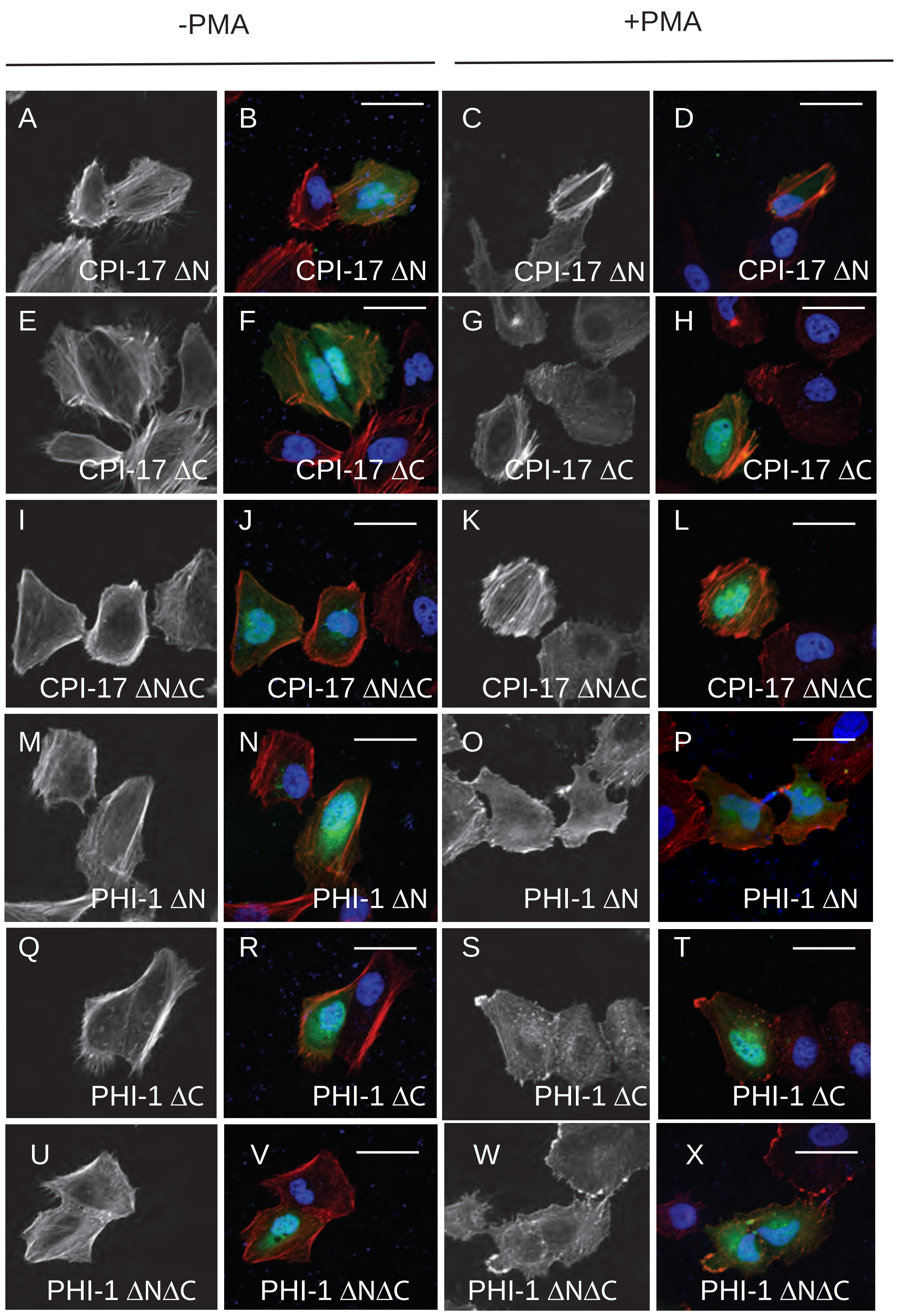

Figure S5. Chimera A but not Chimera B is sufficient to induce stress fiber formation in HeLa cells. HeLa cells were transfected with either GFP-Chimera A (A-D) or GFP-Chimera B (E-H). Cells were treated with either 0.1% DMSO (A, B, E, F) or 1 μM PMA (C, D, G, H) for 3 hours prior to fixation. All cells were fixed and stained with DAPI and Alexa 568-phalloidin to stain the nucleus and actin cytoskeleton, respectively, and imaged with confocal microscopy. Black and white images show phalloidin staining, while color images are a merge of DAPI (blue), GFP (green), and phalloidin (red). White bar indicates 20 μm. Representative cells are shown from a minimum of three biological replicates.

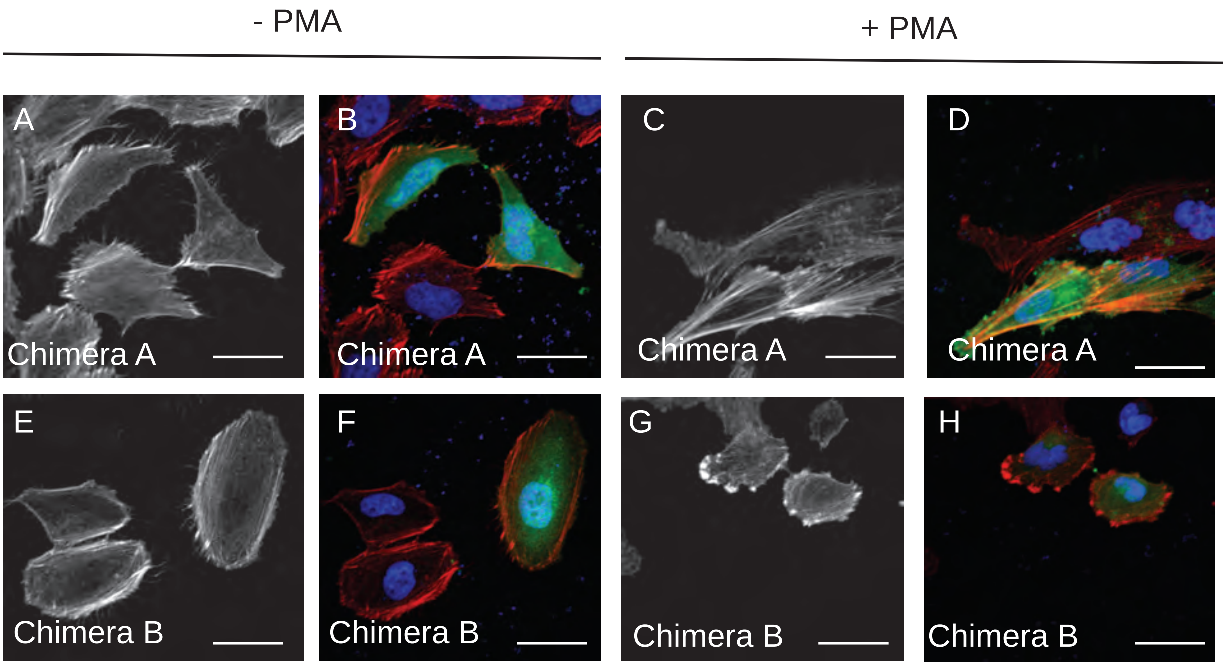

Figure S6. Quantification of stress fiber phenotypes. A., B., C. Images used in Figures S3, S4 and S5 were compiled and scored for stress fiber phenotype. All experiments were replicated 3 to 5 times with the total n in parenthesis, and the data are reported as the percent of total cells imaged. Cells were scored normal if they had numerous mostly parallel stress fibers that cross through the cytoplasm. Excessive actomyosin contractility was scored when numerous focused stress fibers intersect near the cellular nucleus.

A

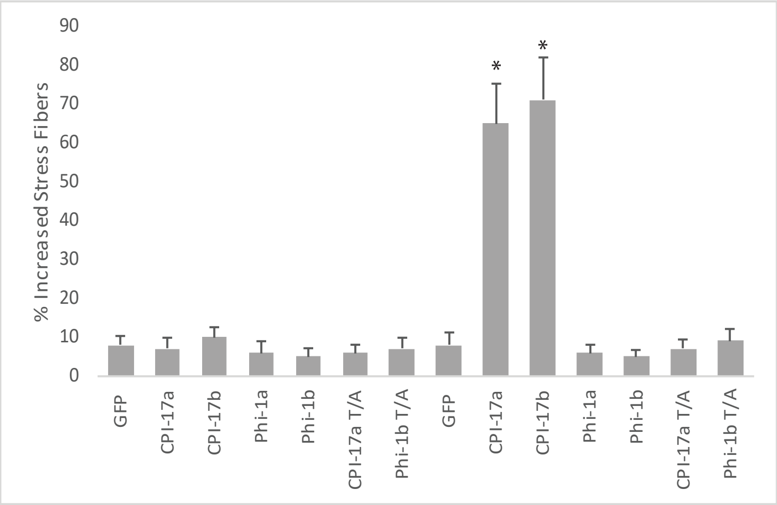

B

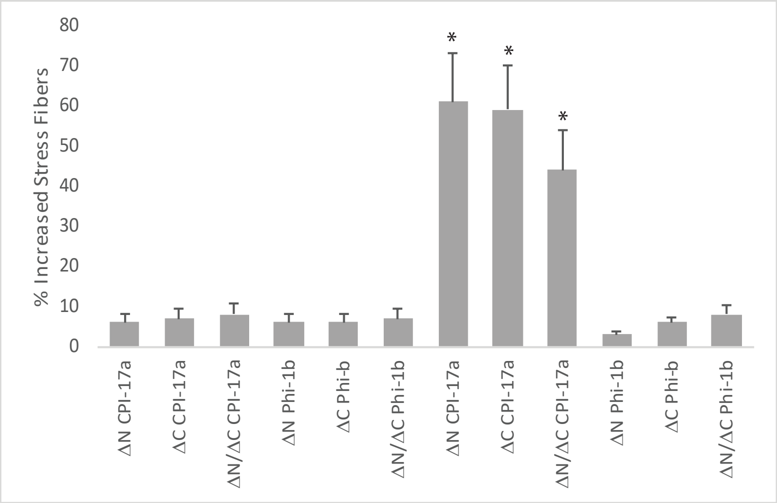

C

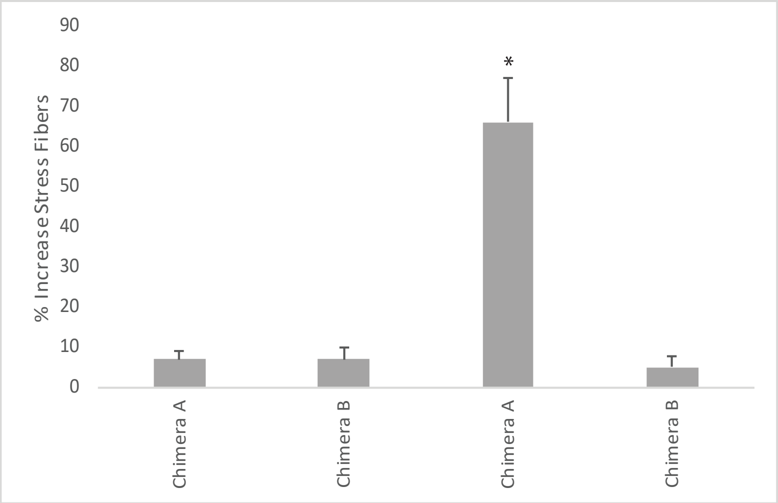

Figure S7. Overexpression of Cpi-17 family members in zebrafish results in gastrulation defects. Quantification of convergent extension defects at bud stage. Each injection was performed a minimum of three times and the percentage of embryos displaying a gastrulation defect is reported. The total number of injected embryos analyzed is reported as n.

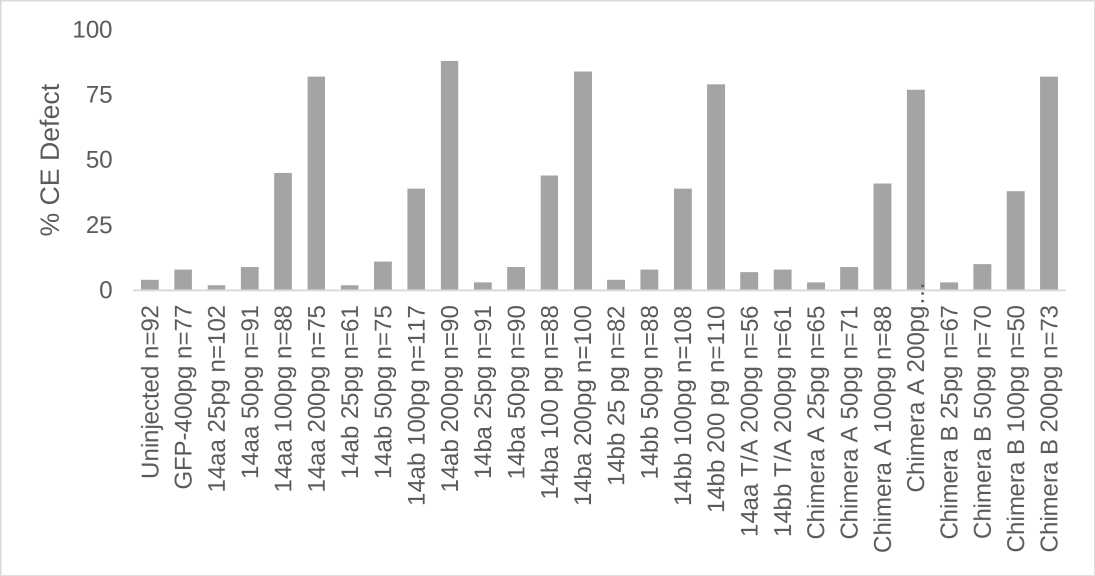

Table S1. Sequences used in this study

Chromosomal loci for each gene in zebrafish

ppp1r14aa	Chromosome 5: 36,130,573-36,139,356 forward strand
ppp1r14ab	Chromosome 15: 20,268,896-20,297,373 reverse strand
ppp1r14ba	Chromosome 21: 26,959,581-26,974,142 reverse strand
ppp1r14bb	Chromosome 7: 60,054,110-60,091,341 forward strand
ppp1r14c	Chromosome 23: 39,914,946-39,956,244 reverse strand
Ppp1r14d	Scaffold KN149966.1: 78,036-78,221 reverse strand

Cpi-17 family sequences used in Figure 1

Danio rerio ppp1r14aa	NP_001017863.1
Danio rerio ppp1r14ab	NP_957104.1
Homo sapiens ppp1r14a	NP_150281.1
Danio rerio ppp1r14ba	NP_001039318.1
Danio rerio ppp1r14bb	NP_001002692.1
Homo sapiens ppp1r14b	NP_619634.1
Danio rerio ppp1r14c	NP_001025134.1
Homo sapiens ppp1r14c	NP_112211.1
Danio rerio ppp1r14d	NP_001082974.1
Homo sapiens ppp1r14d	NP_060196.1

Cpi-17 sequences used in figure S1

Fish species	Accession number
Astyanax mexicanus ppp1r14aa	XP_007257527.1
Danio rerio ppp1r14aa	NP_001017863.1
Cyprinus carpio ppp1r14aa	XP_018932439.1

Sinocylocheilus rhinocerous ppp1r14aa	XP_016430549.1
Lepisosteus oculatus ppp1r14a	XP_015196431.1
Takifugu rubripes ppp1r14aa	XP_003970776.1
Sinocyclocheilus grahamai ppp1r14aa	XP_016084671.1
Tetraodon nigroviridis ppp1r14aa	Not in NCBI, but included in Ensembl tree ENSTNIT00000017474.1
Gasterosteus aculeatus ppp1r14aa	Not in NCBI, but included in Ensembl tree ENSGACT00000026698.1
Oreochromis niloticus ppp1r14aa	XP_003449848.1
Oryzias latipes ppp1r14aa	XP_020564292.1
Poecilia formosa ppp1r14a	XP_007556506.1
Xiphophorus maculatus ppp1r14a	XP_005807158.1
Astyanax mexicanus ppp1r14ab	XP_007246037.1
Danio rerio ppp1r14ab	NP_957104.1
Tetraodon nigroviridis ppp1r14ab	CAG02121.1
Salmo salar ppp1r14aa1	XP_013992601.1
Oncorhynchus mykiss ppp1r14aa2	XP_021473427.1
Oncorhynchus kisutch ppp1r14aa1	XP_020341346.1
Oncorhynchus mykiss ppp1r14aa1	XP_021479539.1
Oncorhynchus kisutch ppp1r14aa2	XP_020320008.1
Oreochromis niloticus ppp1r14ab	XP_019202593.1
Salmo salar ppp1r14aa2	XP_014053165.1
Ictalurus punctatus ppp1r14ab	XP_017345811.1
Cyprinus carpio ppp1r14ab	XP_018935960.1
Stegastes partitus ppp1r14aa	XP_008295588.1
Esox lucius ppp1r14aa	XP_010863938.1

Pygocentrus nattereri ppp1r14ab	XP_017550297
Labrus bergylta ppp1r14aa	XP_020484953.1
Ictalurus punctatus ppp1r14aa	XP_017315399.1
Neolamprologus brichardi ppp1r14aa	XP_006808664.1
Esox lucius ppp1r14ab	XP_010892136.1
Nothobranchius furzeri ppp1r14a	XP_015816111.1
Kryptolebias marmoratus ppp1r14a	XP_017291895.1
Austrofundulus limnaeus ppp1r14a	XP_013879738.1
Oncorhynchus kisutch ppp1r14ab2	XP_020363330.1
Salmo salar ppp1r14ab1	XP_014017643.1
Hippocampus comes ppp1r14ab	XP_019716936.1
Poecilia latipinna ppp1r14a	XP_014889233.1
Scleropages formosus ppp1r14a	XP_018598154.1
Oncorhynchus kisutch ppp1r14ab1	XP_020358477.1
Salmo salar ppp1r14ab2	XP_014069555.1
Oncorhynchus mykiss ppp1r14ab	XP_021443213.1
Haplochromis burtoni ppp1r14a	XP_005936057.1
Cynoglossus semilaevis ppp1r14a	XP_008330726.1
Boleophthalmus pectinirostris ppp1r14a	XP_020790258.1
Cyprinodon variegatus ppp1r14a	XP_015242124.1
Paralichthys olivaceus ppp1r14a	XP_019940965.1
Monopterus albus ppp1r14a	XP_020469069.1
Lates calcarifer ppp1r14aa	XP_018548063.1
Poecilia reticulata ppp1r14a	XP_008426566.1
Larimichthys crocea ppp1r14ab	XP_010746294.2

Larimichthys crocea ppp1r14aa	XP_019123354.1
Pygocentrus nattereri ppp1r14aa	XP_017580470.1
Latimeria chalumnae ppp1r14a	XP_005991672.1
Fundulus heteroclitus ppp1r14aa	XP_021170723.1
Hippocampus comes ppp1r14aa	XP_019713340.1
Stegastes partitus ppp1r14ab	XP_008286242.1
Lates calcarifer ppp1r14ab	XP_018560501.1
Sinocyclocheilus grahami ppp1r14ab	Homologous region of XP_016142379.1
Sinocyclocheilus anshuiensis ppp1r14ab	Homologous region of XP_016308323.1
Sinocyclocheilus rhinocerous ppp1r14ab	Homologous region of XP_016394944.1
Maylandia zebra ppp1r14aa	Homologous region of XP_014263866.1
Pundamilia nyererei ppp1r14aa	Homologous region of XP_005740185.1

PHI-1 sequences used in Figure S2

Fish species	Accession numbers
Danio rerio ppp1r14ba	NP_001039318.1
Lepisosteus oculatus ppp1r14b	XP_015194315.1
Sinocyclocheilus rhinocerous ppp1r14ba	XP_016428675.1
Astyanax mexicanus ppp1r14ba	XP_007244564.1
Cyprinus carpio ppp1r14ba	XP_018919424.1
Pygocentrus nattereri ppp1r14ba	XP_017554273.1
Sinocyclocheilus anshuiensis ppp1r14ba	XP_016317082.1
Sinocyclocheilus grahami ppp1r14ba	XP_016084358.1
Ictalurus punctatus ppp1r14ba	XP_017348815.1
Clupea harengus ppp1r14ba	XP_012695581.1
Boleophthalmus pectinirostris ppp1r14b	XP_020793411.1

Scleropages formosus ppp1r14b	XP_018592966.1
Nothobranchius furzeri ppp1r14b	XP_015802031.1
Takifugu rubripes ppp1r14bb	XP_003966944.1
Kryptolebias marmoratus ppp1r14b	XP_017283465.1
Oreochromis niloticus ppp1r14bb	XP_003452464.1
Lates calcarifer ppp1r14b	XP_018546676.1
Pygocentrus nattereri ppp1r14bb	XP_017555144.1
Paralichthys olivaceus ppp1r14b	XP_019951155.1
Hippocampus comes ppp1r14b	XP_019711667.1
Cyprinodon variegatus ppp1r14b	XP_015229332.1
Monopterus albus ppp1r14b	XP_020465827.1
Clupea harengus ppp1r14bb	XP_012676115.1
Cynoglossus semilaevis ppp1r14b	XP_008317102.1
Astyanax mexicanus ppp1r14bb	XP_007242105.1
Ictalurus punctatus ppp1r14bb	XP_017328504.1
Cyprinus carpio ppp1r14bb	XP_018933598.1
Sinocyclocheilus grahami ppp1r14bb	XP_016124950.1
Esox lucius ppp1r14bb	XP_010903328.1
Sinocyclocheilus anshuiensis ppp1r14bb	XP_016307279.1
Latimeria chalumnae ppp1r14b	XP_005988895.1
Danio rerio ppp1r14bb	NP_001002692.1
Sinocyclocheilus rhinocerous ppp1r14bb	XP_016385272.1
Salmo salar ppp1r14bb1	XP_014061865.1
Oncorhynchus mykiss ppp1r14bb	XP_021447501.1
Oncorhynchus kisutch ppp1r14bb	XP_020330845.1
Salmo salar ppp1r14bb2	XP_013978872.1

Notothenia coriiceps ppp1r14b	XP_010772677.1
Notothenia coriiceps ppp1r14b	XP_010772677.1
Oncorhynchus mykiss ppp1r14ba	XP_021446876.1
Oncorhynchus kisutch ppp1r14ba2	XP_020319545.1
Salmo salar ppp1r14ba1	XP_013984622.1
Oncorhynchus kisutch ppp1r14ba1	XP_020321772.1
Salmo salar ppp1r14ba2	XP_014051938.1
Esox lucius ppp1r14ba	XP_010898826.1

Table S2. Primers used in this study	
qPCR	
ppp1r14aa-For	ATGGCTGAGGAGACACATAC
ppp1r14aa-Rev	GTTCAGCAGATTAACCTTCAC
ppp1r14ab-For	CTGGAGATGGCTGCGAATC
ppp1r14ab-Rev	AATACACCTGTTCACTGAAGG
ppp1r14ba-For	ATGGCAGCGGTAACGAGTC
ppp1r14ba-Rev	CAAGCCAAGTCACGTGGTC
ppp1r14bb-For	ATGGCGGCGGTAACAAGTC
ppp1r14bb-Rev	TCCATGTGTTCATTGAGACAG
Full-length GFP-fusions	
ppp1r14aa-For-EcoRi	AAAGAATTCCATGGCTGAGGAGACACATACCGGTCATTC
ppp1r14aa-Rev-Sali	TATGTCGACGTCACAGTGTCTGATGTGCTGGATGGTGA
ppp1r14ab-For	GAGAATTCATGGCTGCGAATCGGG
ppp1r14ab-Rev-Sali	CTGTCGACCTGCTCAGTGTATCTC
ppp1r14ba-For	GGAGAATTCATGGCAGCGGTAACGAGTC
ppp1r14ba-Rev	GTGTCGACCTGTGGTGAACTCATGGCG
ppp1r14bb-For-EcoRi	AAAGAATTCATGGCGGTAACAAGTCCAGAATCG
ppp1r14bb-Rev-Sali	TATGTCGACTTCATTTCTTCTGAGGAGTACTGAGC
Truncations	
ppp1r14aa-dN-EcoRi	AAGAATTCCATAAGCGCCATTCGCGAGTT
ppp1r14aa-dC-Sali	AAGTCGACTTACTGTTTGTGGAGACCATG
ppp1r14bb-dN-EcoRi	AAGAATTCGTCCGCAAGCAAGGACGGGTC
ppp1r14bb-dC-Sali	AAGTCGACTTACTTCTGCATGCCACGGAT
Chimeras	
Chimera A For	GCTCGAGAGCACGGGATGAATCTAGTCCGCAAGCAAGGACGGGTC
Chimera A Rev	GACCCGTCCTTGCTTGCGGACTAGATTCATCCCGTGCTCTCGAGC
Chimera B For	ATGCTCAATTCCTTCATTCTGTAGTTCCTTCTGCATGCCACGGATCCT
Chimera B Rev	AGGATCCGTGGCATGCAGAAGGAACTACAGAATGAAGGAATTGAGCAT
Full-length His-tag fusions	
ppp1r14aa-For-BglII	GAAGGATCCATGGCTGAGGAGACACATACCGGTCATTCC
ppp1r14ab-For-BamHI	GAGGATCCATGGCTGCGAATCGGG
ppp1r14ba-For-BamHI	GGAGGATCCATGGCAGCGGTAACGAGTC
ppp1r14bb-For-BamHI	AAAGGATCCATGGCGGTAACAAGTCCAGAATCG

pCS2+ full length	
ppp1r14aa-For-ClaI	AAATCGATACCACCATGGCTGAGGAGACACATACCGGTCATTC
ppp1r14aa-Rev-EcoRi	TTGAATTCGTCACAGTGTCTGATGTGCTGGATGGTGA
ppp1r14bb-For-ClaI	AAATCGATACCACCATGGCGGCGGTAACAAGTCCAGAATCG
ppp1r14bb-Rev-EcoRI	TTGAATTCTTCATTTCTTCTGAGGAGTACTGAGC
Mutagenesis	
ppp1r14aa T/A For	CATAAGCGCCATTCGCGAGTTGCTGTTAAATACAACCGCAAACAG
ppp1r14aa T/A Rev	CTGTTTGCGGTTGTATTTAACAGCAACTCGCGAATGGCGCTTATG
ppp1r14bb T/A For	GTCCGCAAGCAAGGACGGGTCGCAGTGAAATATGACCGGAAAGAA
ppp1r14bb T/A Rev	TTCTTTCCGGTCATATTTCACTGCGACCCGTCCTTGCTTGCGGAC
